# A Comprehensive Theory for Relativistic Polaritonic Chemistry: A Four-Component Ab Initio Treatment of Molecular Systems Coupled to Quantum Fields

**DOI:** 10.1021/jacsau.5c00233

**Published:** 2025-07-24

**Authors:** Guillaume Thiam, Riccardo Rossi, Henrik Koch, Leonardo Belpassi, Enrico Ronca

**Affiliations:** † Dipartimento di Chimica, Biologia e Biotecnologie, Università degli Studi di Perugia, Via Elce di Sotto, 8, 06123 Perugia, Italy; ‡ Department of Chemistry, 8018 Norwegian University of Science and Technology, 7491 Trondheim, Norway; § 9327 Istituto di Scienze e Tecnologie Chimiche “Giulio Natta” del CNR(CNR-SCITEC), Via Elce di Sotto, 8, 06123 Perugia, Italy

**Keywords:** polaritonic chemistry, relativistic quantum chemistry, Dirac–Hartree–Fock, ab initio

## Abstract

We present an ab initio approach to study molecules containing heavy atoms strongly interacting with quantum fields in optical devices. The theory has been derived from relativistic quantum electrodynamics (QED), introducing the approximations needed to provide a formalism suitable for relativistic quantum chemistry. This framework represents the ideal starting point to extend the main quantum chemistry methods to relativistic polaritonic. In this article, we present the polaritonic Dirac–Hartree–Fock (Pol-DHF) approach based on this theory. The Pol-DHF approach allows for the simulation of field-induced effects on the ground- and excited-state properties of heavy transition metal molecular complexes. The method is able to include not only the effects of the photons but can in principle be extended also to include explicit interactions with positrons. Application of Pol-DHF to three metal hydrides shows that the magnitude of both polaritonic and relativistic effects can be comparable when relativistic effects become more important. Due to an accurate description of spin–orbit coupling, the method is able to reproduce polaritonic effects happening at the crossing between singlet and triplet potential energy surfaces.

## Graphical abstract

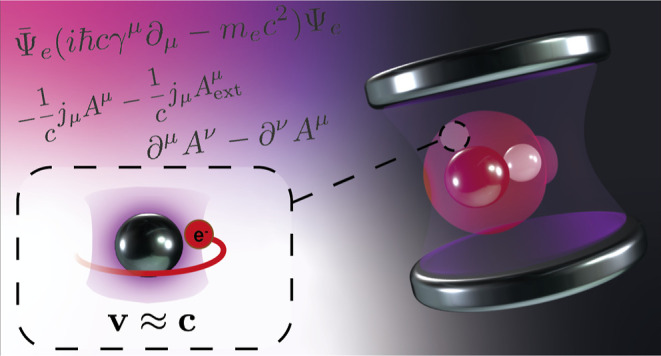

## Introduction

The use of light as a new tool to control and manipulate noninvasively the properties of molecules and materials is opening, in recent years, a new field of research at the border between physics, chemistry, and material science.^1–5^ When matter strongly couples to photons, new hybrid states (polaritons), having partial light and partial matter character, are formed. The strong coupling (SC) condition is usually reached inside properly designed optical devices. The simplest example is the Fabry–Pérot cavity^6^ (Figure 1), made of two highly reflective planar mirrors that confine the photons, leading to a significant enhancement of the light-matter coupling. The cavity frequency and, therefore, the polariton properties can be controlled by changing the geometry and the materials in the device. Several manifestations of polaritonic effects on different physical properties such as absorption spectra, photochemical reaction rates, and conductivity have already been observed in the experiments.^2,3,5,7–10^ What is probably the most well-known demonstration of such effects was obtained in the experiments performed by Ebbesen’s group. In particular, they evidenced that strong coupling to molecular vibrations can be used to catalyze, slow down, or even induce selectivity in chemical reactions.^5,11,12^ These observations opened a new field that is now known as polaritonic chemistry.^13^

**Figure 1:**
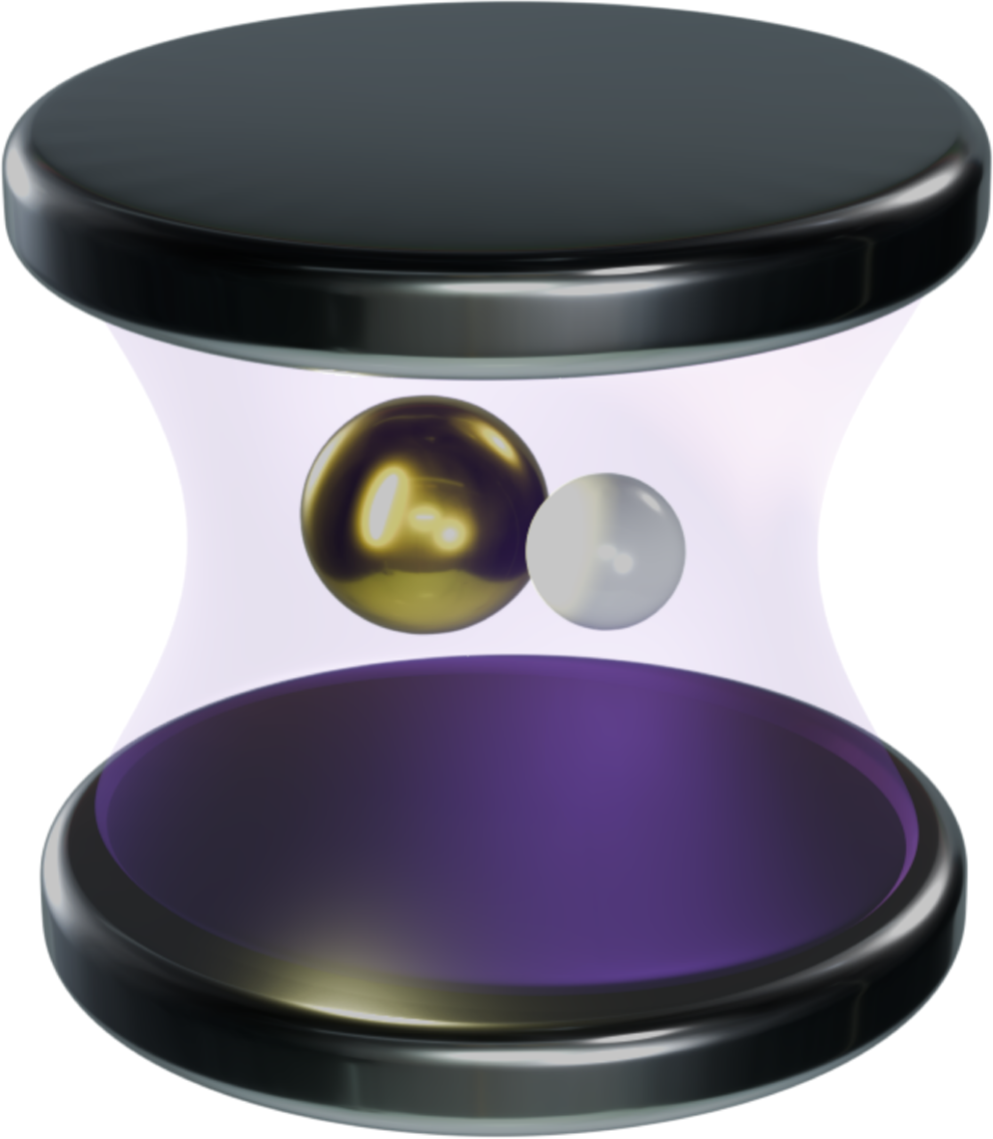
Schematic representation of a Fabry–Pérot cavity containing a gold complex.

In these experiments, the photonic states are usually coupled to either electronic or vibrational states of the molecular systems. However, the electromagnetic nature of the field also allows for modifications of their magnetic properties if one exploits the coupling to spin states. In this way, fine control of the magnetizability and aromatic properties of molecules,^14^ of spin qubits,^15,16^ and of spin phases of materials^17,18^ can be obtained. However, reaching the strong coupling condition in this frequency range is impossible using a simple Fabry–Pérot cavity that, in this case, would require a spacing of centimeters between the mirrors. The problem can be circumvented using planar superconducting devices commonly used in circuit quantum electrodynamics (QED) experiments.^19^ Using similar devices, Affronte’s group has been able to manipulate the spin properties of a single-molecule magnet.^16,20^ Such an accomplishment unlocked many potential applications in spin-qubit-based quantum computation.

In spite of the many improvements in the fabrication of more effective optical devices and the impressive accuracy reached^21,22^ by polaritonic chemistry experiments, many fundamental aspects still remain to be understood. In this context, theory represents a fundamental tool to gain insights into the underlying physics of these processes. In recent years, many ab initio methods, able to treat electron–electron and electron–photon correlation at different levels of accuracy, have been developed.^23–29^ However, they have mainly been applied to investigate cavity-induced effects on the electronic and vibrational degrees of freedom of molecular systems. Only very recently, Barlini et al. proposed the first Hartree–Fock-based approach to study photon-induced effects on the electronic and nuclear magnetic properties of a molecular system.^14^

Spin–orbit coupling and, to some extent, magnetic properties can be seen as a manifestation of relativistic effects in molecules. Therefore, an accurate investigation of these properties requires the inclusion of relativistic effects at different levels of accuracy. This becomes particularly crucial when, like in Affronte’s experiments,^16,20,30–34^ single-molecule magnets containing lanthanide atoms need to be used to have sufficiently long-lasting magnetizations.

Moreover, as already discussed in refs 28 and 35 for Van-der Waals interactions, the quantum electromagnetic field can sometimes enhance small effects that are usually negligible in the absence of photons. From these considerations arises the need for a consistent relativistic quantum electrodynamical ab initio theory able to include all the necessary effects. Formulating such a general approach is one of the goals of this paper. Similar intent was already presented by Ruggenthaler et al.^23^ in a density functional theory (DFT) framework, but only the nonrelativistic limit of the method was actually turned into a useable quantum chemistry implementation. Very recently, Konecny et al.^36^ proposed a relativistic response theory to investigate electronic excitations of relativistic molecules in optical cavities. Though this last approach is able to capture interesting effects like cavity-induced singlet–triplet interactions, at the moment, the implemented approach does not account for modifications induced by the field on the system’s electronic structure. This can be of some importance when investigating molecular properties such as the ionization potential/electron affinity.^25^

In this paper, we develop the first wave function-based relativistic ab initio method describing the ground state of molecular systems coupled to photons in optical cavities. We start the development from the QED Lagrangian to then propose a general relativistic formalism. The latter represents the starting point for the development of mean-field but also, in the future, exact 2-component and correlated methodologies able to perform accurate simulation of these complex systems. We believe that this represents a step forward in the study of polaritonic effects that could have repercussions in various areas of science such as quantum computing, OLED technologies, biochemistry, etc.

The paper is structured as follows: first, a generic derivation of a relativistic QED Hamiltonian theory will be presented, starting from the standard QED Lagrangian. A lot of care has been dedicated to present the theory in a clear and understandable way, even for a nonexpert audience. Then, the Coulomb gauge Hamiltonian, after application of the dipole approximation, is used to develop the first relativistic polaritonic Dirac–Hartree–Fock (Pol-DHF) approach. In this context, some space has been dedicated to the strategies used to deal with negative energy states. Lastly, Pol-DHF has been applied to investigate the electronic properties of small diatomic molecules containing heavy atoms. We end the paper with conclusions and perspectives.

## Theory

In this section, we follow the formal derivation of relativistic QED theory usually presented in physics text books^37,38^ to develop a Hamiltonian formalism that can be applied to formulate new ab initio methodologies for the simulation of polaritonic molecular systems. This choice is meant to render the overall discussion accessible to a broad chemistry audience. The proposed methodology is then used to develop the first Hartree–Fock (HF)-based approach for relativistic molecular systems strongly coupled to quantum fields. For convenience reasons, Gaussian units[Fn fn1] will be used during the whole derivation unless specified otherwise.

In order to develop a quantum theory (see refs 37 and 38 and Section S1.1 of the Supporting Information for more details), one generally needs to start with a Lagrangian formalism from which the fields conjugate momenta can be extracted. Our starting point is the Lagrangian density $L_{QED}=L_{Maxwell}+L_{Dirac}+L_{\mathrm{int}}$ , which contains three distinct contributions. One is purely related to electromagnetism (originating from the Maxwell Lagrangian), one purely describes noninteracting Fermions (originating from the Dirac Lagrangian), and finally, a coupling term allows us to describe interacting Fermions (see Section S1.1 in Supporting Information for more details). From this Lagrangian density, we have constructed the QED Hamiltonian by performing a Legendre transform:

1 $$H_{QED}=\Pi_{\mu }{Ȧ}^{\mu }+\pi {\mathrm{\Psi ̇}}_{e}-L_{QED}$$

where μ ∈ {0, 1, 2, 3} and the Einstein summation convention applies, and repeated indices are summed. The conjugate momenta are given by

2 $$\pi =\frac{\partial L_{QED}}{\partial \dot{\Psi_{e}}}\;\Pi_{\mu }=\frac{\partial L_{QED}}{\partial \dot{A^{\mu }}}$$

the latter are not only necessary to build the Hamiltonian density but also to build the (anti)­commutation relationship needed for the quantum theory. The classical Hamiltonian then reads as

H=−18π∫[−ϕ∇2ϕ︸Elong2−Ȧ·Ȧc2−(∇×A)·(∇×A)︸Etrans.2+B2]dr+∫Ψe†[cαi(−iℏ∇i−ecAi−ecAiext)+βmec2)]Ψedr+∫ϕρdr+∫ϕextρdr. 3

In eq 3, *i* ∈ {1, 2, 3}. $\alpha_{i}=\left(\begin{array}{cc} 0 & \sigma_{i}\\ \sigma_{i} & 0 \end{array}\right)$ and $\beta =\left(\begin{array}{cc} 1 & 0\\ 0 & -1 \end{array}\right)$ are 4 × 4 matrices.

σ_*i*_ are the Pauli matrices:

4 $$\sigma_{x}=\left(\begin{array}{ll} 0 & 1\\ 1 & 0 \end{array}\right)\;\sigma_{y}=\left(\begin{array}{cc} 0 & -i\\ i & 0 \end{array}\right)\;\sigma_{z}=\left(\begin{array}{cc} 1 & 0\\ 0 & -1 \end{array}\right)$$

The auxiliary fields ϕ and **A** are, respectively, the scalar and vector potentials. The latter are not uniquely defined, and many potentials lead to the same electric and magnetic field. This is referred to as gauge freedom^37,39^ (see Section S1.1 of the Supporting Information for more details). In eq 3, no gauge choice has been applied so far. For quantum chemistry applications, the Coulomb gauge (∇·**A** = 0) is the natural choice. The latter allows splitting of the electric field into a longitudinal and a transversal component. This then gives the instantaneous Coulomb interaction. Therefore, in the following, the Coulomb gauge will be adopted. In such a framework, ϕ is related to the electrostatic component of the electric field and to the longitudinal part of the electric field. On the other hand, **A** is related to the magnetic field and to the transverse component of the electric field. We remind the reader that the electric and magnetic fields can be expressed in terms of scalar and vector potentials as $E=-\nabla \phi -\frac{1}{c}\partial_{t}A$ and **B** = ∇ × **A**, respectively. In the Coulomb Gauge, the longitudinal part of the field only depends on ϕ. Under this condition, the Gauss law for the electric field (∇·**E** = 4πρ, where ρ is the electron density) becomes the Poisson’s equation (−∇^2^ϕ = 4πρ) and therefore

5 $$\frac{1}{8\pi }\int dr\phi \nabla^{2}\phi =-\frac{1}{2}\int dr\phi \rho \left(r,t\right)$$

Collecting this term with ∫ϕρ*d* **r** in eq 3 gives the well-known instantaneous electron–electron Coulomb repulsive contribution $\frac{1}{2}\int dr\int dr^{\prime }\frac{\rho_{e}\left(r,t\right)\rho_{e}\left(r^{\prime },t\right)}{\left|r-r^{\prime }\right|}$ .

To facilitate the quantization (see the next section), it is usually convenient to express Hamiltonian 3 in terms of so-called normal variables. These variables are defined in a similar way to the “ladder operator” method used to solve the quantum harmonic oscillator.^37,40^ To begin with, we rewrite Maxwell’s equation in reciprocal space in the following way:

6 $$\partial_{t}E_{trans.}\left(k,t\right)=ick\times B\left(k,t\right)-4\pi J_{trans.}\left(k,t\right)$$

7 $$\partial_{t}B\left(k,t\right)=-ik\times E_{trans.}\left(k,t\right)$$

From the previous equation, one notices the following relationship when $J_{trans.}=0$ :

8 $$\partial_{t}\left(E_{trans.}\pm c\kappa \times B\right)=\pm i\omega \left(E_{trans.}\pm c\kappa \times B\right)$$

where ω = *c*|**k**| and $\kappa =\frac{k}{\left|k\right|}$ . From the previous equation, it appears natural to introduce two new variables, even if $J_{trans.}\neq 0$ :

9 $$\mathrm{\zeta ⃗}\left(k,t\right)=-i\frac{\sqrt{2\pi \hbar \omega }}{2}\left[E_{trans.}\left(k,t\right)-\kappa \times B\left(k,t\right)\right]$$

10 $${\mathrm{\zeta ⃗}}^{*}\left(-k,t\right)=-i\frac{\sqrt{2\pi \hbar \omega }}{2}\left[E_{trans.}\left(k,t\right)+\kappa \times B\left(k,t\right)\right]$$

Using the previous relationship, one is able to express the electric and magnetic fields in terms of normal variables:

11 $$E_{trans.}=i\sqrt{2\pi \hbar \omega }\left(\mathrm{\zeta ⃗}\left(k,t\right)-{\mathrm{\zeta ⃗}}^{*}\left(-k,t\right)\right)$$

12 $$B_{trans.}=i\sqrt{2\pi \hbar \omega }\left(\kappa \times \mathrm{\zeta ⃗}\left(k,t\right)+\kappa \times {\mathrm{\zeta ⃗}}^{*}\left(-k,t\right)\right)$$

**E**_trans._ and **B** can be obtained using a Fourier transform.

Using these normal variables, the Parseval–Plancherel identity^37^ allows writing the following:

13 $$\frac{1}{8\pi }\int d^{3}r\left(E_{trans.}^{2}+B^{2}\right)=\frac{1}{8\pi }\int dk\left({\left|E_{trans.}\right|}^{2}+{\left|B\right|}^{2}\right)$$

and changing **k** → – **k** in the second term of the right-hand side of eq 11, we can rewrite the electromagnetic field Hamiltonian as

14 $$\frac{1}{8\pi }\int d^{3}r\left(E_{trans.}^{2}+B_{trans.}^{2}\right)=\int d^{3}k\frac{\hbar \omega }{2}\left[{\mathrm{\zeta ⃗}}^{*}\mathrm{\zeta ⃗}+\mathrm{\zeta ⃗}{\mathrm{\zeta ⃗}}^{*}\right]$$

where the short notation ζ⃗=ζ⃗(k,t) has been used.

The vector potential **A** can be expressed in terms of the normal variables

15 $$A\left(r,t\right)=\int d^{3}k\sqrt{\frac{2\pi \hbar c}{\omega {\left(2\pi \right)}^{3}}}\left(\mathrm{\zeta ⃗}\exp\left(ik\cdot r\right)+{\mathrm{\zeta ⃗}}^{*}\exp\left(-ik\cdot r\right)\right)$$

Substituting eqs 14 and 15 in eq 3, we obtain the following expression for the classical Hamiltonian:

H=∫d3kℏω2[ζ⃗*ζ⃗+ζ⃗ζ⃗*]+∫drΨe†[cαi(−iℏ∇i−ecAi−ecAexti)+βmec2]Ψe+12∫dr∫d3r′ρe(r,t)ρe(r′,t)|r−r′|+∫ϕextρdr 16

### Hamiltonian Quantization

From now on, the Schrödinger picture is adopted, and therefore, all operators will be considered time-independent. A quantized form of Hamiltonian 16 can be obtained by promoting the normal variables ( ${\mathrm{\zeta ⃗}}^{*}$ /ζ⃗) to the corresponding **k**-dependent field operators ( $a_{\mathrm{\epsilon ⃗}}^{\prime †}$ / $a_{\mathrm{\epsilon ⃗}}^{\prime }$ ), where ϵ⃗ is the polarization vector, exploiting the decomposition of ${\mathrm{\zeta ⃗}}^{*}$ /ζ⃗ on the basis described by both polarization vectors $\mathrm{\zeta ⃗}=\sum_{\epsilon }\zeta_{\epsilon }\mathrm{\epsilon ⃗}$ and ${\mathrm{\zeta ⃗}}^{*}=\sum_{\epsilon }\zeta_{\epsilon }^{*}\mathrm{\epsilon ⃗}$ .

17 $$\begin{array}{l} \zeta_{\mathrm{\epsilon ⃗}}\left(k,t\right)\to a_{\mathrm{\epsilon ⃗}}^{\prime }\left(k\right)\\ \zeta_{\mathrm{\epsilon ⃗}}^{*}\left(k,t\right)\to a_{\mathrm{\epsilon ⃗}}^{\prime †}\left(k\right) \end{array}$$

satisfying the following commutation relations:

18 $$\left[a_{\mathrm{\epsilon ⃗}}^{\prime }\left(k\right),a_{{\mathrm{\epsilon ⃗}}^{\prime }}^{\prime †}\left(k^{\prime }\right)\right]=\delta_{\mathrm{\epsilon ⃗}{\mathrm{\epsilon ⃗}}^{\prime }}\delta \left(k-k^{\prime }\right)$$

In terms of the field operator, the vector potential becomes

19 A(r)=∑ϵ→∫d3k2πℏcω(2π)3[aϵ⃗′(k)exp(ik·r)ϵ⃗+aϵ⃗′†(k)exp(−ik·r)ϵ⃗]

In the confined space of the optical cavity, the wave vector **k** assumes discrete values, consequently defining a discrete spectrum of field modes characterized by the direction of the **k** vector and by the polarization of the field oscillations (ϵ⃗) . Ideally, one should consider the full sum and include an infinite amount of modes; however, it is in practice unfeasible. Therefore, quite often, only one or a few modes are included explicitly, while the rest are simply not considered. This approximation is usually acceptable if the molecular states are well separated compared to the magnitude of the Rabi splitting (cf Figure 2). In the relativistic context, the single-/few-mode approximation can also be applied, but it requires a little more attention on the selection of the modes that need to be selected/discarded. There, we will identify the mode enhanced by the device (e.g., cavity, circuit, plasmon, etc.) as **k**_dm_. Doing so, we can rewrite the vector potential in such a way:

20 $$A\left(r\right)=A_{dm}\left(r\right)+A_{om}\left(r\right)$$

where

21 Adm(r)=∑ϵ→A(kdm)[aϵ⃗′(kdm)exp(ikdm·r)ϵ⃗+aϵ⃗′†(kdm)exp(−ikdm·r)ϵ⃗].

and

22 $$A_{om}\left(r\right)=A\left(r\right)-A_{dm}\left(r\right)$$

where $A\left(k_{dm}\right)=\sqrt{\frac{2\pi \hbar c}{\omega_{dm}{\left(2\pi \right)}^{3}}}$ . Splitting the sum into two terms allows for the identification of the terms corresponding to the mode enhanced by the device and **A**_om_ corresponding to the remaining part of the vector potential containing all of the other modes. Such a splitting of vector potential **A** is motivated by the fact that the ∫*d* **rj**·**A** term, where **j** = *ec*Ψ_*e*_^†^αΨ_*e*_, includes several effects. In particular, it includes at the same time the electron–photon interaction as well as many other known energy terms (current–current term, frequency-dependent Breit term, vacuum polarization, etc.^38,41,42^ ). Such a rewriting allows us to handle separately the photons coupled to the molecular system due to the device and what are known as relativistic corrections. Doing so, we can exploit tools from both ab initio polaritonic quantum chemistry and relativistic quantum chemistry to evaluate the various contributions. Since the molecular systems are usually significantly smaller than the wavelength of the cavity field, we are entitled to apply the dipole approximation, imposing that exp (*i* **k**_dm_·**r**) ∼1 and $A_{dm}\left(r\right)∼A_{dm}(0⃗)$ in eq 20. This significantly simplifies the photonic part of the Hamiltonian.

**Figure 2:**
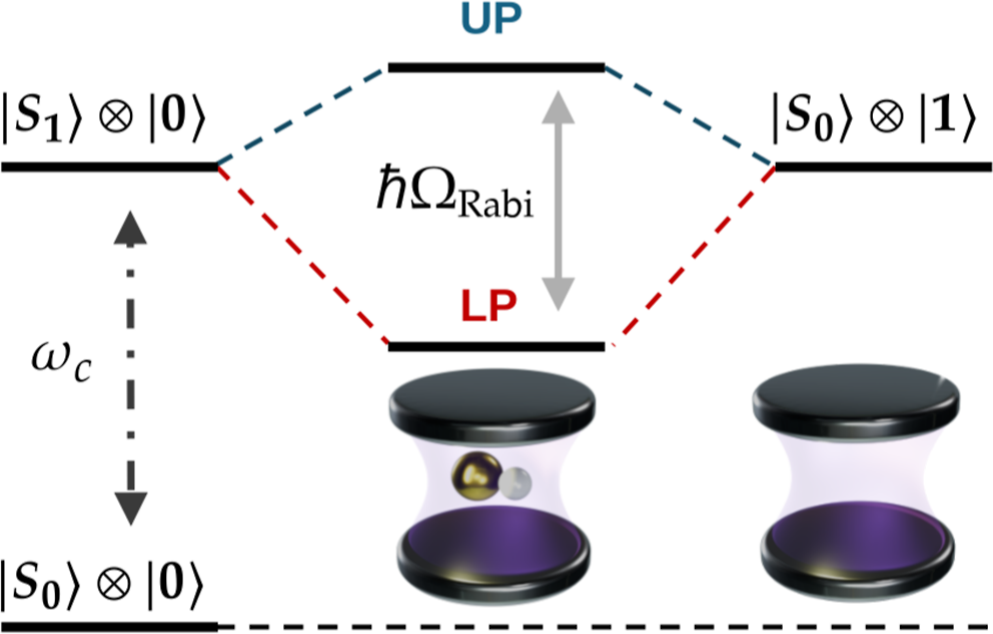
Scheme of a generic Rabi splitting (ℏΩ_Rabi_). UP and LP indicate the upper and lower polaritons, respectively.

For the Fermionic part, the spinor fields are promoted to spinor field operators satisfying the following equal-time anticommutation relations:

23 $$\left\{\Psi {\left(\mathrm{r⃗}\right)}_{\mu },\Psi^{†}{\left({\mathrm{r⃗}}^{\prime }\right)}_{\nu }\right\}=\delta_{\mu \nu }\delta \left(\mathrm{r⃗}-{\mathrm{r⃗}}^{\prime }\right)$$

The QED Hamiltonian can then be written as

24 HQED=∫d3Ψ†hD(1)Ψ+12∫drd3r′Ψ†(r)Ψ(r)1|r−r′|Ψ†(r′)Ψ(r′)+∑ϵ→ℏωdm,ϵ⃗(adm,ϵ⃗′†adm,ϵ⃗′+12)

where

hD(1)=cαi(pi−ecAdm,i(0⃗)−ecAom,i(r)−ecAext,i(r))+ϕext+βmec2. 25

Since we assume the single-mode approximation, the radiative term $\left(\sum_{\vec{\epsilon }}\hbar \omega_{om,\mathrm{\epsilon ⃗}}\left(a_{om,\mathrm{\epsilon ⃗}}^{\prime †}a_{om,\mathrm{\epsilon ⃗}}^{\prime }+\frac{1}{2}\right)\right)$ involving the other modes only contributes in a trivial way. Therefore, in order to make the equations easier to read, we discarded this term, which only represents a mere rigid shift of the energy levels of the system.

### Relativistic Pauli–Fierz Hamiltonian in the Length Gauge

When investigating molecular systems, it is usually more convenient to apply a unitary transformation to the field modes, allowing for direct coupling between the field operators and the molecular dipole. This transformation is known as the length-gauge transformation:

26 $$U=\exp\left(\frac{ie}{\hbar c}A_{dm}(0⃗)\cdot R\right)$$

where **R** = ∫*d* **r**Ψ^†^ **r**Ψ. Hamiltonian 24 can be transformed into the length-gauge form by the application of eq 26:

27 $$H_{RPF}^{l}=U^{†}H_{QED}U$$

This step must be followed by two other transformations: first, a rotation which ensures that the photonic part is real, and then a so-called coherent state transformation to remove the apparent origin dependence in the Hamiltonian^23,24,43^ (more details on the derivation can be found in Section S1.3 in Supporting Information and in ref 44). It is important to point out that the requirement for the photonic part to be real is not crucial in the relativistic case, but, for consistency with the nonrelativistic works, we chose to keep it also in this context.

Using these transformations, we obtain

28 HRPFl=∫drΨ†hΨ+12∫drdr′Ψ†(r)Ψ(r)1|r−r′|Ψ†(r′)Ψ(r′)+∑ϵ→ℏωdm(aϵ⃗†aϵ⃗+12)+(ec2πVϵ⃗·[R−⟨R⟩])2−ℏωdm(ec2πVϵ⃗·[R−⟨R⟩])(aϵ⃗+aϵ⃗†)

Here, $a_{dm,\mathrm{\epsilon ⃗}}$ and $a_{dm,\mathrm{\epsilon ⃗}}^{†}$ have been relabeled to emphasize that, due to the length gauge transformation, the latter are modified. In fact, $\left\langle a_{dm,\mathrm{\epsilon ⃗}}^{†}a_{dm,\mathrm{\epsilon ⃗}}\right\rangle$ does not coincide with the number of photons related to the mode **k**_dm_ anymore.^45^ In eq 28, the presence of the expectation^46–49^ value of the total dipole moments (*e*⟨**R**⟩) ensures that the Hamiltonian will not explicitly depend on the origin of the reference system for neutral molecules. In atomic and molecular physics/chemistry, it is usually more convenient to expand the electronic field on the basis of atomic/molecular orbitals (MOs), which are solutions of the Dirac equation in an external potential (the Coulomb potential of the nuclei, for instance). The field operator expanded on such a basis writes as

29 $${\mathrm{\Psi ̂}}_{e}\left(\mathrm{r⃗}\right)=\sum_{p}{ĉ}_{p}\phi_{p}\left(\mathrm{r⃗}\right)\;\text{and}\;{\mathrm{\Psi ̂}}^{†}\left({\mathrm{r⃗}}^{\prime }\right)=\sum_{p}{ĉ}_{p}^{†}\phi_{p}^{†}\left(\mathrm{r⃗}\right)$$

where the hat is used to indicate operators. ϕ_*p*_ is the solution of the Dirac equation with an external potential. Notice that ϕ are monoelectronic/positronic spinors. In the following, the hat will be dropped to avoid an overload of symbols. The electronic creation (*c* ^†^) and annihilation (*c*) operators satisfying anticommutation rules:

30 $$\left\{c_{p},c_{q}^{†}\right\}=\delta_{pq}$$

Using these operators, the energy components of Hamiltonian 28 can be expressed in a second quantized form implementable in a quantum chemistry code. In the following, we will use a shorthand notation for the mono- and bi-electronic integrals that are defined as follows:

31 $$O_{pq}=\int dr\phi_{p}^{†}\left(r\right)O\phi_{q}\left(r\right)$$

32 $$\left(pq|rs\right)=\int drdr^{\prime }\phi_{p}^{†}\left(r\right)\phi_{r}^{†}\left(r^{\prime }\right)g\left(r,r^{\prime }\right)\phi_{s}\left(r^{\prime }\right)\phi_{q}\left(r\right)$$

where *O* is a generic one-body operator and (*pq*|*rs*) are the well-known two-electron integrals. Therefore, the second quantized (sq) Hamiltonian is

33 $$H_{RPF}=H^{\left(1\right)}+H^{\left(2\right)}+H^{\left(3\right)}$$

where

34 H(1)=∑pq[hpq+2πe2Vc2(Qpq−2⟨ϵα·R⟩(ϵα·rpq))]︸h̃pqcp†cqH(2)=12∑pqrs[(pq|rs)+2×2πe2Vc2(ϵα·rpq)(ϵα·rrs)]︸(pq|~rs)cp†cr†cscqH(3)=∑ϵ→ℏωdm(Nϵ⃗+12)+2πe2Vc2⟨ϵ⃗·R⟩2

where *Q* refers to the molecular quadrupole and $N_{\mathrm{\epsilon ⃗}}=a_{\mathrm{\epsilon ⃗}}^{†}a_{\mathrm{\epsilon ⃗}}$ .

As previously mentioned, the interaction term ∫*d* **rj**·**A**_om_ has to be evaluated in an approximated way. Currently, a lot of effort from the relativistic quantum chemistry community is devoted to developing accurate and efficient ways to evaluate the contributions emerging from such a term.

An extensive summary about how to include these effects can be found in refs 38, 42 , and^46–49^ . In bound state QED (BSQED), this term is evaluated using techniques from quantum field theory (QFT), which, however, represents tremendous effort. Such a formalism, while providing remarkable accuracy for atomic systems, is not really applicable to molecular electronic structure calculations. Another strategy is the use of effective potentials (Breit potential,^38,50,51^ Uehling,^38,42,50,52^ Wichmann–Kroll^42,53^ potential, and self-energy contributions^38,42,54,55^ ). Such a strategy has been rather successful in evaluating full-Breit contribution and QED corrections with a satisfying accuracy. This approach represents the most convenient strategy to be adopted in order to compare these effects to the polaritonic ones. At this point, we emphasize that such effects are fairly small, and Gaunt/Breit and QED corrections are mostly important for core orbitals where polaritonics mostly influence the valence orbitals. Nonetheless, in some cases, their effect on molecular properties such as ionization potential or electron affinity can be of comparable magnitude (∼0.01–0.05 eV).^25,56,57^ This point might require some carefulness and should be further studied in the future. Notice that Hamiltonian 33 allows for both positive and negative energy states. This point will be discussed in detail in the next section.

### Treatment of the Negative Energy States

Physically, the negative energy solutions correspond to positronic states.^41^ This reinterpretation becomes clearer if we split the sum over all states into a sum over the positive energy states and another over the negative energy ones. In this picture, the negative energy electron creation (annihilation) operators are reinterpreted as positive energy positron annihilation (creation) operators (see refs 41 and 58–60 ). In this framework, the field operator can be decomposed in this way:

35 $$\Psi_{e}\left(\mathrm{r⃗}\right)=\sum_{p}\left\{c_{p}\phi_{p}\left(\mathrm{r⃗}\right)+b_{p}^{†}\psi_{-p}\left(\mathrm{r⃗}\right)\right\}$$

36 $$\Psi_{e}^{†}\left(\mathrm{r⃗}\right)=\sum_{p}\left\{c_{p}^{†}\phi_{p}^{†}\left(\mathrm{r⃗}\right)+b_{p}\psi_{-p}^{†}\left(\mathrm{r⃗}\right)\right\}$$

37 $$\left\{c_{p},c_{q}^{†}\right\}=\delta_{pq}\;\text{and}\;\left\{b_{p},b_{q}^{†}\right\}=\delta_{pq}$$

where *b*_*p*_ and *b*_*p*_^†^ are respectively positron annihilation and creation operators. Using these operators, Hamiltonian 33 can be rewritten in terms of both electronic and positronic contributions:

38 $${\mathrm{H̅}}_{RPF}={\mathrm{H̅}}^{\left(1\right)}+{\mathrm{H̅}}^{\left(2\right)}+H^{\left(3\right)}$$

where

39 $${\mathrm{H̅}}^{\left(1\right)}=\sum_{p,q}\left\{c_{p}^{†}c_{q}{\mathrm{h̃}}_{pq}+c_{p}^{†}b_{q}^{†}{\mathrm{h̃}}_{p\mathrm{q̅}}+b_{p}c_{q}{\mathrm{h̃}}_{\mathrm{p̅}q}-b_{q}^{†}b_{p}{\mathrm{h̃}}_{\mathrm{p̅}\mathrm{q̅}}\right\}$$

40 H̅(2)=12∑p,q,r,s{cp†cr†cscq(pq|~rs)+2(pq|~rs̅)cp†cr†bs†cq+2(pq|~r̅s)cp†brcscq−2(pq|~r̅s̅)cp†bs†brcq+2(pq̅|~r̅s)cp†bq†brcs+(pq̅|~rs̅)cp†cr†bs†bq†+(p̅q|~r̅s)bpbrcscq−2(p̅q̅|~r̅s)bq†bpbrcs−2(p̅q̅|~rs̅)cr†bs†bq†cp+(p̅q̅|~r̅s̅)bs†bq†bpbr}

H^(3)^ and the modified one- and two-electron integrals in eq 38 have been defined in eq 34. Barred indices correspond to positronic indices. In this article, we do not address the role of the Fermionic vacuum that is at the heart of many discussions.^58–60^ Indeed, the choice of the Hamiltonian and the possible reinterpretation of negative energy states have an influence on the nature of the Fermionic vacuum, which in turn has some consequence on the expectation value of the Hamiltonian. Fortunately, at the Hartree–Fock level, there is no real dependence on the vacuum nature (however, it becomes important when developing correlated methods).

### The Hartree–Fock Approximation

Since it treats explicitly all the interactions between relativistic electrons/positrons and the photons of the cavity field, Hamiltonian 38 represents the perfect starting point for the development of ab initio theories suitable to simulate molecular systems containing heavy atoms in optical devices. In this section, we will use Hamiltonian 38 to develop the first relativistic 4-component Hartree–Fock (HF) approach for polaritonic chemistry. In quantum chemistry, HF represents the simplest approximation, respecting the right symmetry of all the involved particles. HF also provides access to physically meaningful sets of atomic and molecular orbitals that can be subsequently used to develop more accurate correlated theories. In the present paper, ϕ_ext_ has been replaced by the (point charge) Coulomb potential of the nuclei in the Born–Oppenheimer approximation and **A**_ext_ = 0 in Hamiltonian 38. In future work, the point charge nuclear potential will be replaced by a finite-size model, but from a formal point of view, it does not introduce any major change to the equations. The two-electron integrals correspond to the instantaneous Coulomb interaction. HF is a mean field theory; hence, the involved quantum species do not directly interact with each other but travel in space under the action of the average potential of the other particles. For the purely electronic case, this implies that the wave function is described by a single Slater determinant of atomic/molecular orbitals or spinors in the relativistic case:

41 $$\Phi \left(r_{1},...,r_{N}\right)=\frac{1}{\sqrt{N!}}\left|\begin{array}{cccc} \phi_{1}\left(r_{1}\right) & \phi_{1}\left(r_{2}\right) & \cdots & \phi_{1}\left(r_{N}\right)\\ \phi_{2}\left(r_{1}\right) & \phi_{2}\left(r_{2}\right) & \cdots & \phi_{2}\left(r_{N}\right)\\ \vdots & \vdots & \ddots & \vdots \\ \phi_{N}\left(r_{1}\right) & \phi_{N}\left(r_{2}\right) & \cdots & \phi_{N}\left(r_{N}\right) \end{array}\right|$$

This ansatz ensures the antisymmetry of the wave function under particle exchange. In this case, three kinds of particles are involved: electrons, positrons, and photons, each one respecting its own statistics (Fermionic for electrons/positrons and bosonic for photons). The wave function can be written as the product of distinct wave functions for every species:
42

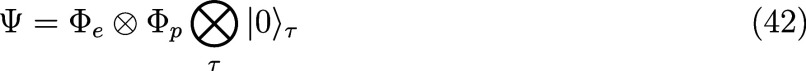

where Φ_*e*_ and Φ_*p*_ are single determinants for electronic and positronic spinors, respectively, |0⟩_τ_ is the vacuum state associated with the photonic mode τ=(k,ϵ⃗) , and ⊗_τ_|0⟩_τ_ is a shorthand notation for ∏_τ_ ⊗|0⟩_τ_. A similar ansatz has already been applied in the nonrelativistic version of the QED-HF approach, and its implications are discussed in detail in ref 24. Ansatz 42 can be rewritten, for practical reasons, in terms of second-quantized electronic and positronic operators as
43

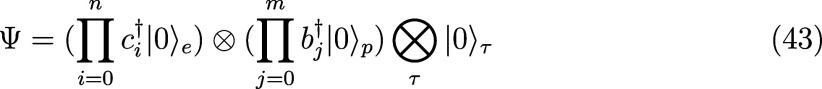

where |0⟩_*e*_ and |0⟩_*p*_ are the electronic and positronic vacuum states, respectively.

Projecting Hamiltonian 38 on eq 42, we can calculate the relativistic polaritonic HF energy:

⟨E⟩DHF=∑ph̃pp−∑p®h̃p̅p̅+12∑p,q{(pp|~qq)−(pq|~qp)}+12∑p̅,q̅{(p̅p̅|~q̅q̅)−(p̅q̅|~q̅p̅)}−∑p,q̅{(pp|~q̅q̅)−(pq̅|~q̅p)}+∑αℏωα(Nα+12)+⟨hnuc⟩+2πe2Vc2⟨ϵα·R⟩2 44

where

45 $${\mathrm{h̃}}_{pp}=h_{pp}-\frac{2\pi e^{2}}{Vc^{2}}\left[Q_{pp}-2\left\langle \epsilon_{\alpha }\cdot R\right\rangle \left(\epsilon_{\alpha }\cdot r_{pp}\right)\right]$$

46 $${\mathrm{h̃}}_{\mathrm{p̅}\mathrm{p̅}}=h_{\mathrm{p̅}\mathrm{p̅}}-\frac{2\pi e^{2}}{Vc^{2}}\left[Q_{\mathrm{p̅}\mathrm{p̅}}-2\left\langle \epsilon_{\alpha }\cdot R\right\rangle \left(\epsilon_{\alpha }\cdot r_{\mathrm{p̅}\mathrm{p̅}}\right)\right]$$

47 $$\left(pp|\sim qq\right)=\left(pp|qq\right)-2\times \frac{2\pi e^{2}}{Vc^{2}}\left(\left(\epsilon_{\alpha }\cdot r_{pp}\right)\left(\epsilon_{\alpha }\cdot r_{qq}\right)\right)$$

48 $$\left(pq|\sim qp\right)=\left(pq|qp\right)-2\times \frac{2\pi e^{2}}{Vc^{2}}\left(\left(\epsilon_{\alpha }\cdot r_{pq}\right)\left(\epsilon_{\alpha }\cdot r_{qp}\right)\right)$$

49 $$\left(\mathrm{p̅}\mathrm{p̅}|\sim \mathrm{q̅}\mathrm{q̅}\right)=\left(\mathrm{p̅}\mathrm{p̅}|\mathrm{q̅}\mathrm{q̅}\right)-2\times \frac{2\pi e^{2}}{Vc^{2}}\left(\left(\epsilon_{\alpha }\cdot r_{\mathrm{p̅}\mathrm{p̅}}\right)\left(\epsilon_{\alpha }\cdot r_{\mathrm{q̅}\mathrm{q̅}}\right)\right)$$

50 $$\left(\mathrm{p̅}\mathrm{q̅}|\sim \mathrm{q̅}\mathrm{p̅}\right)=\left(\mathrm{p̅}\mathrm{q̅}|\mathrm{q̅}\mathrm{p̅}\right)-2\times \frac{2\pi e^{2}}{Vc^{2}}\left(\left(\epsilon_{\alpha }\cdot r_{\mathrm{p̅}\mathrm{q̅}}\right)\left(\epsilon_{\alpha }\cdot r_{\mathrm{q̅}\mathrm{p̅}}\right)\right)$$

51 $$\left(pp|\sim \mathrm{q̅}\mathrm{q̅}\right)=\left(pp|\mathrm{q̅}\mathrm{q̅}\right)-2\times \frac{2\pi e^{2}}{Vc^{2}}\left(\left(\epsilon_{\alpha }\cdot r_{pp}\right)\left(\epsilon_{\alpha }\cdot r_{\mathrm{q̅}\mathrm{q̅}}\right)\right)$$

52 $$\left(p\mathrm{q̅}|\sim \mathrm{q̅}p\right)=\left(p\mathrm{q̅}|\mathrm{q̅}p\right)-2\times \frac{2\pi e^{2}}{Vc^{2}}\left(\left(\epsilon_{\alpha }\cdot r_{p\mathrm{q̅}}\right)\left(\epsilon_{\alpha }\cdot r_{\mathrm{q̅}p}\right)\right)$$

Notice that at the HF level, all terms that do not conserve the number of particles will have a zero expectation value. This is the case for the so-called bilinear term in eqs 28, which involves only one annihilation (creation) operator and therefore changes the number of photons. Consequently, this term gives a zero contribution to the ground-state energy.

#### The Fock Operator

As for the bare electron case, the HF problem can be solved by minimizing eq 44 for variations in the spinors. This can be done by performing a rotation of the monoelectronic/positronic orbitals using the following operator:

53 $$K=\sum_{pq}K_{pq}c_{p}^{†}c_{q}$$

and then imposing the stationary condition:

54 $$\frac{\partial {\left\langle E\right\rangle }_{DHF}}{\partial K_{pq}}=0$$

From this minimization, we can obtain, following standard orbital rotation techniques,^51,58,61^ a new Fock operator with matrix elements:

55 fpq=h̃pq+∑k[(pq|~kk)−(pk|~kq)]−∑k®[(pq|~k̅k̅)−(pk̅|~k̅q)]

where *p* and *q* are either positron or electron indices.

The Fock operator in eq 55 can be applied in a Roothan–Hall-like procedure:^61^

56 FC=εC

to optimize the orbital coefficients. It is important to highlight that exactly as in the nonrelativistic QED-HF approach (see ref 24), the Fock matrix in eq 55 suffers from an explicit origin dependence if charged systems need to be investigated. This problem can be solved by applying the so-called strong coupling (SC) HF approximation proposed in ref 27. In this paper, we will focus, for the moment, on neutral molecular systems, and an SC extension of our method will be the topic of future work.

Due to the presence of negative energy states in the Hamiltonian spectrum, the spinor optimization procedure is in fact a minimax problem.^38,51^ In our approach, equivalent results can be obtained when no explicit positron is included in the wave function ansatz:
57

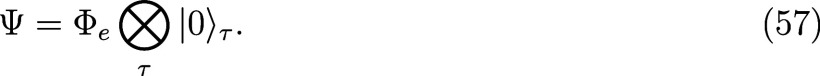

consequently, the projection of Hamiltonian 38 on such wave functions will cancel all positronic-dependent terms in eq 44.

This ansatz is the natural extension of the one usually used in nonrelativistic QED-HF.^24,27^ In this paper, this simplified ansatz will be used to generate the results presented in the Results section. The positronic degrees of freedom will be included explicitly in a future implementation of the method.

#### Kinetic Balance in the Presence of the Field

In relativistic quantum chemistry, the spinors can be expressed as two-component objects:

58 $$\left[\begin{array}{l} \Psi_{1}\\ \Psi_{2}\\ \Psi_{3}\\ \Psi_{4} \end{array}\right]=\left[\begin{array}{l} \Psi^{L}\\ \Psi^{S} \end{array}\right]$$

where Ψ^*L*^ and Ψ^*S*^ are called, respectively, “large” and “small” components. In practice, the spinor solutions are expanded on a basis set: Ψ^*T*^ = ∑_μ_ *C*_μ_^*T*^χ_μ_^*T*^, where *T* = {*L*, *S*} and χ^*T*^ is a 2-spinor. In order to avoid the so-called variational collapse of the solution, a constraint on the large and small components of the basis set is applied.^38,50^ In the presence of a vector potential, such constraint has the following form:

59 $$\chi^{S}=\frac{\sigma \cdot \pi }{2m_{e}c}\chi^{L}$$

where **σ** = (σ_*x*_, σ_*y*_, σ_*z*_) is the vector of the three Pauli matrices and $\pi =p-\frac{e}{c}A$ is the generalized momentum. Such prescription is called the magnetic balance condition.^62–65^ In standard relativistic quantum chemistry, the momentum is simply **p**. We refer to this condition as the kinetic balance.^38,50^ In our case, since the generalized momentum is a priori dependent on the field, the magnetic balance needs, in principle, to be satisfied. This could require having a field-dependent small-component basis set. However, in the present context, we have shown that in the dipole approximation, applying the length gauge transformation, the momentum operator of the theory is thus transformed in the following way (see Section S1.3 of the Supporting Information for a more detailed derivation):

60 $$p-\frac{e}{c}A\left(0⃗\right)\to p$$

Consequently, the kinetic balance condition:

61 $$\chi^{S}=\frac{\sigma \cdot p}{2m_{e}c}\chi^{L}$$

can be applied in this case. It is important to stress that this is only possible under the dipole approximation; otherwise, the length gauge transformation would not provide such a simple expression for the Hamiltonian and the associated momentum.

### Excited-State Properties

In polaritonic chemistry, the signature property emerging from the strong coupling condition is the Rabi splitting. It represents the energy separation between the polaritons formed by the mixing between matter and field states (see Figure 2). Calculating Rabi splittings, which could directly be compared with experimental data, requires access to the excited states of the coupled light–matter system. At the HF level, they can be simulated by recurring to linear response theory. Recently, Castagnola et al.^66^ presented an HF linear response theory for nonrelativistic polaritonic systems. In this section, we present an extension of this approach to 4-component Dirac HF. Using linear response theory, excitation energies (ω_*I*_) can be obtained by solving the well-known Casida equation:^67^

62 $$\left[\begin{array}{ll} A & B\\ B^{*} & A^{*} \end{array}\right]\left(\begin{array}{c} \vec{X_{I}}\\ \vec{Y_{I}} \end{array}\right)=\omega_{I}\left[\begin{array}{cc} +1 & 0\\ 0 & -1 \end{array}\right]\left(\begin{array}{l} \vec{X_{I}}\\ \vec{Y_{I}} \end{array}\right)$$

The polaritonic expressions of the *A* and *B* matrices as derived in ref 66 assume the form

63 $$A=\left[\begin{array}{cc} \omega_{\alpha }\delta_{\alpha \beta } & \sqrt{\omega_{\alpha }}\left(\lambda_{\alpha }\cdot d_{ib}\right)\\ \sqrt{\omega_{\alpha }}\left(\lambda_{\alpha }\cdot d_{bi}^{*}\right) & A_{el} \end{array}\right]$$

64 $$B=\left[\begin{array}{cc} 0 & -\sqrt{\omega_{\alpha }}\left(\lambda_{\alpha }\cdot d_{ib}\right)\\ -\sqrt{\omega_{\alpha }}\left(\lambda_{\alpha }\cdot d_{bi}^{*}\right) & B_{el} \end{array}\right]$$

where **A**_*el*_ and **B**_*el*_ have matrix elements

65 $${\left(A_{el}\right)}_{ia,bj}=\delta_{ij}f_{ab}-\delta_{ab}f_{ij}+2\left(ai|\sim bj\right)-\left(ab|\sim ji\right)$$

66 $${\left(B_{el}\right)}_{ia,bj}=\left(bi|\sim aj\right)-2\left(ai|\sim bj\right)$$

A similar approach has been recently proposed by Konecny et al.^36^ to calculate excitation energies at the QED Dirac–Kohn–Sham level of theory based on previous work presented in refs 68 and 69 .

## Results

### Methods

All the results presented in this section have been obtained from a development version of the PySCF software package.^70,71^ All energies have been converged using default parameters in PySCF. In particular, the Pol-DHF approach has been implemented by applying modifications to the integrals used in standard DHF.^72^ By default the spinor integrals are evaluated on a basis set including both $j=l\pm \frac{1}{2}$ . From a technical point of view, the Pol-DHF method scales as the standard DHF method (they only differ by some prefactor). So far, to simplify the interpretation of the results, we neglected the positrons in the treatment. A detailed discussion of the effects induced by the explicit inclusion of the positronic degrees of freedom will be the topic of a future follow-up paper. All calculations have been performed using the all-electron x2c-SVP basis set^73–76^ for the large component. The small component was obtained via the kinetic balance prescription. The coupling value has been set to 0.05 au. Though this coupling is not accessible with a standard Fabry–Pérot cavity, it can be reached by including collective effects or with other types of devices, as in ref 77. In this study, one molecule coupled to the device is considered to have a high coupling value. However, it should not change the qualitative character of the effects observed.^78^ Moreover, including more molecules in the device can only be meaningful if electron–photon correlation is included in the theory.^25,57^ This will be the topic of follow-up work. The data sets generated and analyzed during the current study can be reproduced using the PySCF development code, the input files, and the geometries provided at the Zenodo link.^79^

### Ground-State Properties

In this section, we analyze the field-induced effects on the ground-state properties of three metal hydrides (CuH, AgH, and AuH). These complexes contain metals belonging to different periods of the 11th group of the periodic table. Going down the groups, the velocity of the electrons (in particular, of the inner ones) increases, approaching finite fractions of the speed of light in the gold case. In fact, gold complexes are well-known to have interesting electronic properties due to the significant relativistic effects.^80–83^ In this paper, we will analyze how the polaritonic effects compete with the relativistic effects, generating modifications of the molecular electronic structure. In Table 1, we compare the total energies calculated at the DHF level of theory with those evaluated including the field (Pol-DHF).

**Table 1:** Ground-State Total Energies Calculated at the DHF and Pol-DHF Levels^a^

molecule	DHF (Hartree)	Pol-DHF (Hartree)	Δ*E*_Pol_ (eV)
CuH	–1653.11275	–1653.11843	–0.1543
AgH	–5338.68917	–5338.69790	–0.2374
AuH	–21639.06979	–21639.07709	–0.1988

a Δ*E*_Pol_ = *E*_Pol‑DHF_ – *E*_DHF._

The field-induced effects, despite being small compared to the total energies, still represent a sizable (some tens of eV) variation in the energy of the system. In particular, it is interesting to notice that the field-induced energy variation is quite similar for copper and gold (Cu, 0.15 eV; Au, 0.20 eV), while it is slightly bigger (in absolute value) for silver (Ag,0.24 eV). This trend can actually be explained by the fact that under the QED-HF approximation, the only field term contributing to the total energy of the system is the dipole self-energy contribution ${\left(\frac{e}{c}\sqrt{\frac{2\pi }{V}}\epsilon_{\alpha }\cdot \left[R-\left\langle R\right\rangle \right]\right)}^{2}$ . As highlighted in eq 34, the one-electron term coming from the dipole self-energy directly depends on the molecular quadrupole, and it is well-known from the literature (see ref 84) that this, as many other electronic properties (atomic radius, etc.),^85^ shows a very similar trend if we move down the group.

The effects observed on the total energy values are an indirect observation of the variations induced by the field on the system’s molecular orbitals (MOs).

In Figure 3, we compare the orbital energies for the valence orbitals (*nd* and (*n* + 1)­sσ) of the three complexes evaluated at the Pol-DHF level with those calculated at the DHF and spin-free eXact-2-component (SFX2C) levels of theory.^70,71,86^ As expected, the biggest variations in the orbital energies can be observed by comparing the spin-free X2C results (as implemented in PySCF) with the DHF ones. In this case, the usual destabilization of the d orbitals and consequent stabilization of the sσ MOs can be observed. The reduction of the gap between these occupied MOs decreases with the increasing relativistic character of the metal (smaller for Au than for Cu). The effect of the field on the electronic structure, though sizable, is much smaller.

**Figure 3:**
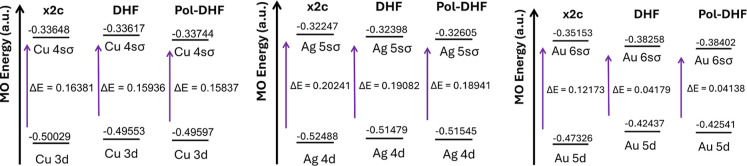
Change in energies from *nd* → (*n* + 1)­sσ in a.u.

The field-induced effects (Δ­(Δ*E*)) on the *nd* → (*n* + 1)­sσ energy gap, barely appreciable in Figure 3, are reported in Table 2. As already observed for the total energy, the largest field-induced variation of the energy gap is observed for AgH. It is crucial to point out that even in the gold case, where the effect is smaller, the cavity field is able to induce a ∼10 meV variation on the orbital gap. These values represent a reasonable fraction (∼0.25 or higher) of a kcal/mol relevant to observing variations of the chemical properties. It is important to highlight that the effects on the ground-state properties we just presented are only accessible if the ultrastrong coupling regime,^77^ like the one we simulated in our test analysis, can be reached. However, it is also important to remind us that the cavity-induced ground-state effects could have a significant impact on the electronic and nuclear spin excitations (usually strongly affected by relativistic effects) also in more moderated coupling regimes. This aspect will be the topic of a following study. Interestingly, the relative change induced by the field $\left(\chi_{E}=\frac{\Delta \left(\Delta E\right)}{\Delta E}\times 100\right)$ is larger for AuH (about 1% of the total gap) compared to the other systems (0.7% for AgH and 0.6% for CuH). This is a small difference, and it is clearly not sufficient to draw conclusions. An extended study to verify if this trend still holds going down the periodic table could yield some interesting insights. Notice that these field-induced variations of the *nd* → (*n* + 1)­sσ energy gap lead (for all systems) to a reduction of the gap due to a very small stabilization of the (*n* + 1)­sσ MOs accompanied by a larger destabilization of the *nd* orbitals.

**Table 2:** First Column is the Difference of the *nd* → (*n* + 1)­sσ Gap in meV^a^

molecule	Δ(Δ*E*) (meV)	χ_E_ (%)
CuH	26.9	0.62
AgH	38.4	0.74
AuH	11.2	0.98

a The second column represents the relative change in energy due to polaritonic effects.

#### Gaunt, Breit, and Polaritonic Contributions to Ground-State Energies

In this work, we were able to include in a variational way the frequency-independent Breit term:

67 $$B\left(i,j\right)=-e^{2}\left\{\underset{Gaunt}{\underset{︸}{\frac{\alpha_{i}\alpha_{j}}{\left|r_{i}-r_{j}\right|}}}+\underset{Breit}{\underset{︸}{\frac{1}{2}\left(\alpha_{i}\cdot \nabla_{i}\right)\left(\alpha_{j}\cdot \nabla_{j}\right)\left|r_{i}-r_{j}\right|}}\right\}$$

and performed a comparison between the energy variations introduced by the quantum field, with the Gaunt and Breit terms.

In Table 3, Gaunt–Breit corrections to the DHF and Pol-DHF energies are presented. These effects are quite sizable, in particular for AuH, which is the system exhibiting the largest relativistic effects. In this case, the trend is monotonic, and both the Gaunt and Breit effects increase going down the group of the periodic table. It is important to notice that if the absolute energy variation is taken into account, these effects are at least 1 order of magnitude larger than the effects generated by the quantum field. The comparison between the Breit correction in CuH (the less relativistic system) and the corresponding polaritonic energy correction, shown in Table 1, demonstrates that quite unequivocally. These observations clearly indicate that for a molecular system coupled to a quantum field, attention needs to be paid to omitting the Breit term. More striking results can be obtained if we perform the same analysis of the orbital energies. In Figure 4, the absolute value of the energy contributions (in logarithmic scale) due to the Gaunt, Breit, and polaritonic terms on the energies of the occupied MOs are presented for the three systems.

**Table 3:** Energy Differences between DHF and DHF–Gaunt­(–Breit) Levels

molecule	no Pol		Pol	
	Gaunt (eV)	Breit (eV)	Gaunt (eV)	Breit (eV)
CuH	20.6381	–1.8419	20.6381	–1.8419
AgH	109.7382	–10.8607	109.7380	–10.8607
AuH	544.0871	–66.5148	544.0874	–66.5149

**Figure 4:**
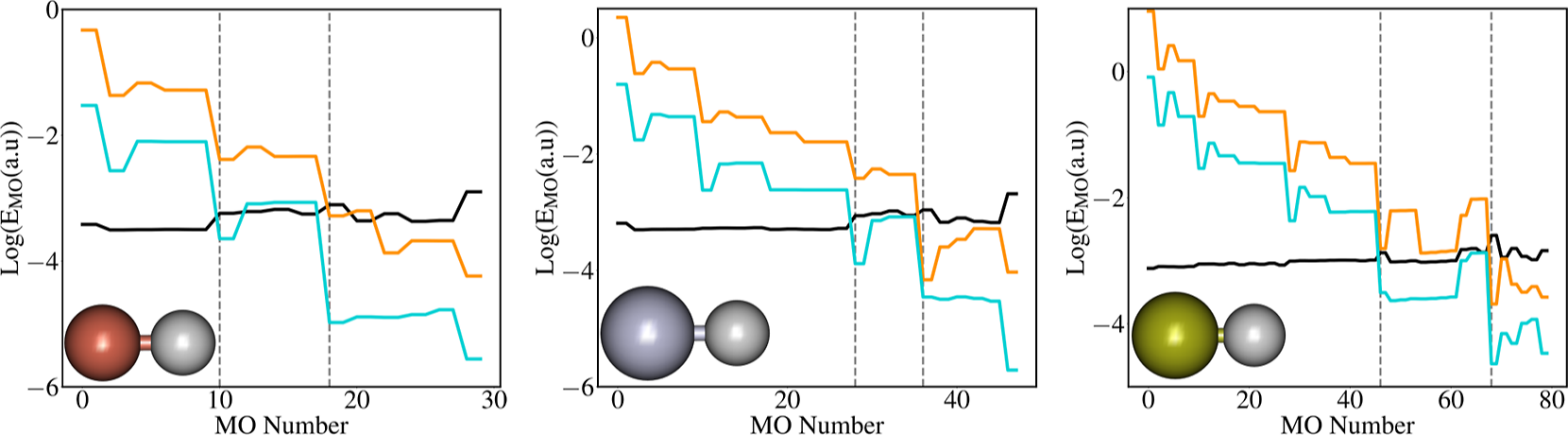
Absolute contribution of polaritonic (black), Gaunt (orange), and Breit (cyan) terms on the various molecular orbitals of CuH (left panel), AgH (middle panel), and AuH (right panel). Three zones have been represented on the graph; the one on the left corresponds to the core region; the middle one is an area where Gaunt, Breit, and Polaritonic contributions are comparable; and the area on the right is the valence region.

The most evident aspect is the very wide variation range of the energy contributions for different molecular orbitals. In general, the effects due to the full-Breit term are much more sizable for the core orbitals, while they monotonically decrease moving toward the valence ones. This trend is clearly expected and has been documented in ref 87. This behavior is obviously more evident for AuH than for CuH. The polaritonic contribution shows, instead, much smaller variations, and the effect slightly increases moving toward the valence. In the core region, the Gaunt and Breit contributions are always larger, while in the valence region, the polaritonic effects are dominant, at least in the coupling regime used in our calculations. Interestingly, for the intermediate orbitals, all of the effects are quite comparable, confirming that the inclusion of the Breit term could induce crucial effects in molecular systems coupled to photons. For example, they might be important if the Rabi splittings for excitations from a core orbital to a valence orbital are being studied or when the induced effect of Gaunt/Breit/polaritonic on IP and EA^25,56,57^ is investigated. However, keep in mind that such a context goes beyond the dipole approximation and the length gauge. However, other strategies have been proposed to circumvent such an issue (see refs 66 and 78 ). Still, there are cases where neglecting such effects, as is usually done in relativistic quantum chemistry, is still reasonable. For instance, if we are interested in excited-state properties (i.e., Rabi splitting, etc.) involving only valence electrons, a phenomenon recently analyzed by Konecny et al. in ref 36, then omitting current–current interaction and the Coulomb gauge correction in the treatment is clearly meaningful. For this reason, in the next section, excited states will be investigated without including the full-Breit correction. However, if excitations from inner orbitals (i.e., core excitations, etc.) need to be analyzed, the inclusion of the full-Breit term will be necessary. Bear in mind that the implications of the effects presented in this section are, in some sense, minimized by the absence of electron–electron and electron–photon correlation in the treatment. For instance, the HF approximation removes the frequency dependence of the ground-state energy from the frequency of the field. This dependence can be recovered only by including correlation into the model.^27^ We expect that the inclusion of frequency-dependent terms will be crucial in particular to describe resonant processes. The inclusion of electron–electron and electron–photon correlation will be the main topic of a future follow-up paper.

### Excited-State Properties

In the previous section, we focused on the field-induced effects on the ground-state properties of three metal hydrides of the 11th group of the periodic table. In this section, we analyze in detail the effects generated by the photons on the optical properties of the AuH complex.^88,89^ Similar analyses for CuH and AgH are presented in Section S2 of the Supporting Information. In this system, because of strong relativistic effects, we expect to observe spectra very different compared to those simulated without taking relativity into account. This is obvious from Figure 5.

**Figure 5:**
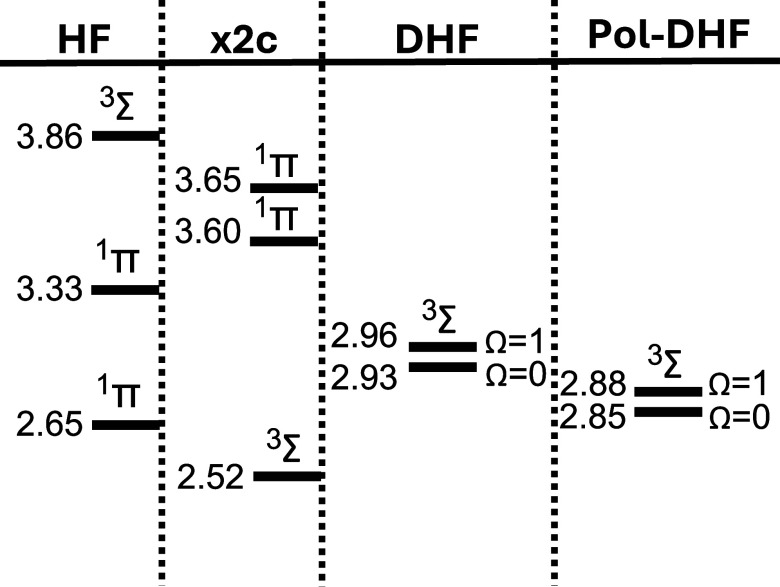
Off-resonance ω = 0 excitation energies for AuH in eV with different levels of calculation.

As can be seen, at the HF level, the triplet state (^3^Σ) is found to be higher in energy than the Π singlet states, in disagreement with the experimental data from refs 88 and 89 . The inclusion of a spin-free relativistic correction partially resolves this issue, but it underestimates the energy by about ∼0.45 eV compared to DHF calculations. DHF does not only reproduce the right ordering of the states, but it also describes the breaking of the degeneracy to form (from the triplets) the Ω = 0 and Ω = 1 states. For an improved readability of the plot in Figure 5, the Pol-DHF data have been calculated off-resonance. This choice highlights the energy shift due to the dipole self-energy. In resonance conditions, the Rabi splitting (discussed later in Figure 6) would also be visible. Comparing this data with the bare electronic DHF ones, we see that the field (in on-resonance conditions) induces a stabilization of the Ω = 0 and Ω = 1 states without inducing any change in the ordering of the states.

**Figure 6:**
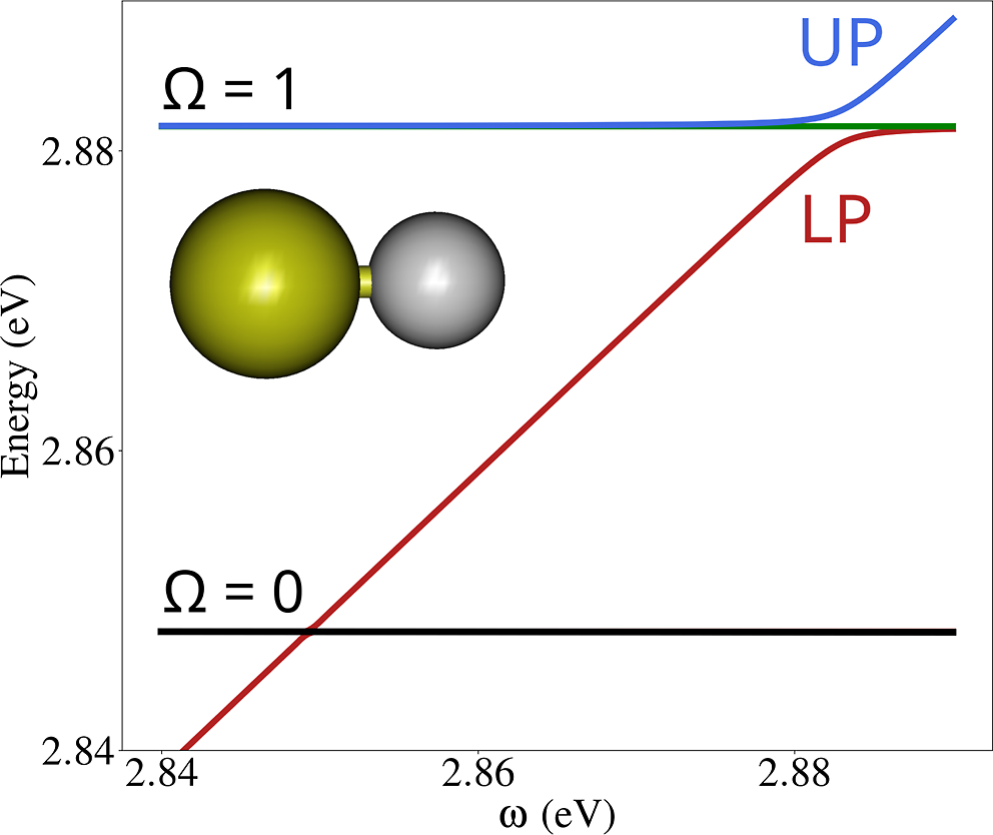
Excitation energies evaluated with linear response Pol-DHF as a function of the cavity frequency for AuH. UP stands for upper polariton and LP for lower polariton.

Finally, in Figure 6, we reported the dispersion of the AuH excitation energies as a function of the cavity frequency. The excitations falling in the investigated energy range refer to the Ω = 0 and Ω = 1 states obtained by the splitting of the triplets. For a nonrelativistic system, we should not observe any Rabi splitting due to the Δ*J* = ± 1 selection rule. In this case instead, due to the strong spin–orbit coupling, a sizable splitting can be observed at the crossing between the first photonic replica of the ground state (^1^Σ) and the Ω = 1 state. For this system, the energy difference between Ω = 0 and Ω = 1, associated with spin–orbit coupling, is 1 order of magnitude larger (0.03 eV) than the Rabi splitting (∼10^–3^ eV). However, a word of caution is necessary. In this work, for computational reasons, we have used a contracted basis set, which is usually not recommended when working in 4-component frameworks. In order to assess the importance of such a choice, we have performed calculations with uncontracted basis sets for all the systems at the ground-state levels and for the excited state of CuH. At the ground-state level, the results are not significantly affected. For the excited-state calculations, for CuH, the use of an uncontracted basis set reduces the magnitude of the Rabi splitting, rendering the latter of comparable size compared to the spin–orbit coupling. This is rather reasonable since the transition is a priori forbidden, and a weak spin–orbit coupling induces a weaker transition dipole moment. Still, even for a system where the spin–orbit coupling is not strong, it appears that polaritonic effects can be of a comparable size (more details are provided in Section S3 of Supporting Information). This spin–orbit coupling-induced singlet–triplet Rabi splitting was already reported for a different system by Konecny et al. in ref 36; though the systems are different, no qualitative differences in behavior are expected. These observations clearly demonstrate how the electromagnetic field can be used to manipulate and control intersystem crossing processes and, consequently, the phosphorescence of complexes containing heavy atoms. In Section S2 of the Supporting Information, a similar discussion is also reported for CuH and AgH.

## Conclusions

In this paper, we proposed a reformulation of relativistic QED allowing for an easier development of ab initio methodologies to simulate heavy atom molecular complexes in strong coupling conditions. Using this theoretical ground, we reported the development and implementation of the first relativistic polaritonic wave function-based ab initio method, namely, Pol-DHF. The theory has been presented starting from the standard Lagrangian and has been derived into a useable implementation of the Pol-DHF code. Considering the possible competition with the polaritonic effects, the importance of radiative QED corrections has been addressed, though their inclusion is left for future work. After providing a roadmap to the implementation, we presented applications of Pol-DHF to three metal hydrides: CuH, AgH, and AuH. These systems were an excellent test case in order to assess the magnitude of the polaritonic effects in comparison to the relativistic effects. To do so, we evaluated the influence of the polaritonic effects on the *nd* → (*n* + 1)­sσ gap for the three systems. The polaritonic effects resulted in having the largest relative influence on AuH, for which relativistic effects are more prominent. Afterward, we provided a detailed analysis of the competition between the Gaunt, Breit, and polaritonic effects on the ground-state and orbital energies. We have verified that in the polaritonic context, even though the full-Breit term represents a significantly larger contribution to the total ground-state energy, its effect remained extremely orbital dependent. In particular, its impact is much stronger on the core orbitals compared to the valence, where instead the polaritonic effects dominate, in particular, if very strong coupling values can be reached. Therefore, we could conclude that neglecting the full-Breit term in strong coupling conditions is not always possible; in particular, it can be done only if properties involving exclusively valence orbitals need to be investigated. A similar study could be conducted adding effective potentials, or using BSQED techniques, to include radiative QED corrections and comparing the magnitude of their effects to the polaritonic ones; this will surely be the topic of a future work. Lastly, we presented excited-state calculations for AuH at the TD-Pol-DHF level. As already observed by Konecny et al.,^36^ we have shown that using a fully relativistic polaritonic theory, the appearance of Rabi splittings at the crossing between singlet and triplet potential energy surfaces can be observed. Furthermore, we have provided a comparison between polaritonic and relativistic effects (e.g., Rabi splitting vs spin–orbit coupling). Lastly, Pol-DHF represents the perfect platform upon which one can develop more elaborate methods (e.g., including dynamic and static correlation). Pol-DHF also opens the door for the development of polaritonic x2c methods and would represent a point of reference that would facilitate such an endeavor. We strongly believe that the methodologies and the applications presented in this paper can represent a significant step toward the simulation of relativistic molecular systems strongly coupled to photons, a field that is recently finding many interesting applications in photochemistry but also in spintronics and quantum computation.

## Supplementary Material



## Data Availability

The development version of PySCF used to produce the data sets presented in this manuscript, together with the input files and the geometries, is available at the Zenodo link.^79^

## Footnotes

a [cm, g, s].

## Notes

Additional theoretical details regarding the derivation of the QED Hamiltonian and the length gauge transformation; excitation energies with respect to the cavity frequency for CuH and AgH; ground-state total energies at the DHF and Pol-DHF levels for CuH using an uncontracted basis set; energy differences between DHF and DHF–Gaunt­(–Breit) levels for CuH with an uncontracted basis set; Gaunt, Breit, and polaritonic contribution in absolute value to the total energy with respect to the MO number for CuH using an uncontracted basis set; and excitation energies with respect to the cavity frequency for CuH using an uncontracted basis set (PDF)
