# Supplementary material for: A Comprehensive Theory for Relativistic Polaritonic Chemistry: A Four-Component Ab Initio Treatment of Molecular Systems Coupled to Quantum Fields
Source: JACS Au. 2025 Jul 24;5(8):3775–88. doi: 10.1021/jacsau.5c00233 (PMC12381748; doi:10.1021/jacsau.5c00233)
Supplement: Supplementary file 1 [file au5c00233_si_001.pdf]

Supporting Information to: "A comprehensive theory for relativistic polaritonic chemistry: a four components ab initio treatment of molecular systems coupled to quantum fields"

Guillaume Thiam,[*,†] Riccardo Rossi,[†] Henrik Koch,[‡] Leonardo Belpassi,[¶] and Enrico Ronca[*,†]

†*Dipartimento di Chimica, Biologia e Biotecnologie, Università degli Studi di Perugia, Via Elce di Sotto, 8,06123, Perugia, Italy*

‡*Department of Chemistry, Norwegian University of Science and Technology, 7491 Trondheim, Norway*

¶*Istituto di Scienze e Tecnologie Chimiche "Giulio Natta" del CNR (CNR-SCITEC), Via Elce di Sotto, 8, 06123 Perugia, Italy*

E-mail: guillaume.thiam@unipg.it; enrico.ronca@unipg.it

Supporting information:

Section 1: Additional theoretical details.

Section 2: Excited States properties of CuH and AgH.

Figure SI1: Excitation energies with respect to the cavity frequency for CuH.

Figure SI2: Excitation energies with respect to the cavity frequency for AgH.

Section 3: Effect of using an uncontracted basis set.

Table SI1: Ground state total energies at the DHF and Pol-DHF level for CuH using an uncontracted basis set.

Table SI2: Energy differences between DHF and DHF-Gaunt(-Breit) level for CuH with an uncontracted basis set

Figure SI3: Gaunt, Breit and Polaritonic contribution in absolute value to the total energy with respect to MO number for CuH using an uncontracted basis set.

Figure SI4: Excitation energies with respect to the cavity frequency for CuH using an uncontracted basis set.

1 Theory derivation

In this section, we follow the formal derivation of relativistic QED theory usually presented in physics text books[1,2] to develop a Hamiltonian formalism that can be applied to formulate new *ab initio* methodologies for the simulation of polaritonic molecular systems. This choice has been meant to render the overall discussion accessible to a broad chemistry audience. The proposed methodology is then used to develop the first Hartree-Fock (HF) based approach for relativistic molecular systems strongly coupled to quantum fields. For convenience reasons, Gaussian units will be used during the whole derivation unless specified otherwise.

1.1 The Quantum Electrodynamics Lagrangian

We start our derivation from the definition of a Lagrangian describing at the same time the relativistic molecular system, the electromagnetic field and their interaction. In the following, all Greek letters indices span the components of 4 indices vectors (from 0 to 3), whereas latin letters only span the spatial components of the vector (from 1 to 3). Moreover, Einstein summation conventions are used.

To describe matter, we use the standard Lagrangian density for Dirac fields:

$$\mathcal{L}_{\text{Dirac}} = \bar{\Psi}_e (i\hbar c\gamma^\mu \partial_\mu - m_e c^2)\Psi_e \tag{1}$$

where c is the speed of light and m_e is the mass of the electron. The matrices γ^μ are defined as:

$$\gamma^0 = \begin{pmatrix} \mathbf{1} & \mathbf{0} \\ \mathbf{0} & -\mathbf{1} \end{pmatrix} \quad \gamma^i = \begin{pmatrix} \mathbf{0} & \sigma^i \\ -\sigma^i & \mathbf{0} \end{pmatrix} \tag{2}$$

with $\boldsymbol{\sigma}^i$ representing the Pauli matrices:

$$\sigma^x = \begin{pmatrix} 0 & 1 \\ 1 & 0 \end{pmatrix} \quad \sigma^y = \begin{pmatrix} 0 & -i \\ i & 0 \end{pmatrix} \quad \sigma^z = \begin{pmatrix} 1 & 0 \\ 0 & -1 \end{pmatrix} \tag{3}$$

The γ^μ matrices are needed to construct Lorentz invariant quantities and naturally include the spin-orbit coupling in the theory. $\partial_\mu = \left(\partial_t, \partial_x, \partial_y, \partial_z\right)$ is the 4-derivative and Ψ_e are the electron spinor fields having $\bar{\Psi}_e = \Psi_e^\dagger \gamma^0$ as adjoint.

The dynamics of the electromagnetic field is described instead by the Lagrangian density ($\mathcal{L}_{\text{Maxwell}}$):

$$\mathcal{L}_{\text{Maxwell}} = -\frac{1}{16\pi} F^{\mu\nu} F_{\mu\nu} \tag{4}$$

where the field tensor $F^{\mu\nu}$:

$$F^{\mu\nu} = \partial^\mu A^\nu - \partial^\nu A^\mu, \tag{5}$$

depends on the 4-vector potential $A^\nu = (\phi, \mathbf{A})$ and mediates all the electromagnetic interactions. In the context of polaritonic chemistry, such a term accounts both for the field induced by the electron and the one inherent to the confinement of the molecular system in the cavity. Maxwell's equations allow to define auxiliary scalar and vector potentials, respectively ϕ and $\mathbf{A}$. These potentials are not uniquely defined, and many potentials lead to the same electric and magnetic field. This is referred to as gauge freedom:[1,3]

$$\mathbf{A}' = \mathbf{A} + \nabla f \tag{6}$$

$$\phi' = \phi - \frac{1}{c}\frac{\partial f}{\partial t} \tag{7}$$

where f is a scalar function. ϕ is related to the electrostatic component of the electric field, and, in Coulomb gauge, to the longitudinal part of the electric field. On the other hand, $\mathbf{A}$ is related to the magnetic field, and, in Coulomb gauge, to the transverse component of the electric field. We remind the reader that the electric field can be expressed in terms of scalar and vector potentials $\mathbf{E} = -\nabla\phi - \frac{1}{c}\partial_t \mathbf{A}$ and the magnetic field in terms of vector potential $\mathbf{B} = \nabla \times \mathbf{A}$. The choice of the Lagrangian density for the electromagnetic field is not unique, and different equivalent forms can be used depending on the gauge. Lagrangian 4 is usually the most convenient choice in Coulomb gauge ($\nabla \cdot \mathbf{A} = 0$[3,4]). This Lagrangian is not always convenient if other gauges[5,6] (e.g. Lorenz gauge[3]) need to be used. The light and matter terms are coupled via interaction contributions:

$$\mathcal{L}_{\text{Int}} = -\frac{1}{c} j_\mu A^\mu - \frac{1}{c} j_\mu A^\mu_{\text{ext}} \tag{8}$$

where A^μ_{ext} is an external four vector potential that can be associated to the nuclei, to an external (non-dynamical) electromagnetic field, etc. At the moment, its definition remain general and will be specified when needed.

The 4-current $j^\mu = (c\rho, \mathbf{j})$ in Eq. 8 can be expressed as a function of Ψ_e and γ^μ as:

$$j^\mu = ec\bar{\Psi}_e \gamma^\mu \Psi_e. \tag{9}$$

Finally, the complete QED Lagrangian takes the form:

$$\mathcal{L}_{\mathrm{QED}} = \mathcal{L}_{\mathrm{Dirac}} + \mathcal{L}_{\mathrm{Maxwell}} + \mathcal{L}_{\mathrm{Int}} \tag{10}$$

Note that only the fermionic (Ψ_e) and electromagnetic fields (A^μ) will be treated as dynamical variables.[2] Eq. 10 will be the starting point for the development of the Hamiltonian formalism derived in the following.

1.2 Hamiltonian formulation in Coulomb gauge

Starting from Lagrangian 10 a Hamiltonian formulation of the theory can be derived by performing a Legendre transform:

$$\mathcal{H}_{\mathrm{QED}} = \Pi_\mu \dot{A}^\mu + \pi \dot{\Psi}_e - \mathcal{L}_{\mathrm{QED}} \tag{11}$$

where the conjugate momenta are given by:

$$\pi = \frac{\partial \mathcal{L}_{\mathrm{QED}}}{\partial \dot{\Psi}_e} \qquad \Pi_\mu = \frac{\partial \mathcal{L}_{\mathrm{QED}}}{\partial \dot{A}^\mu} \tag{12}$$

Substituting Eq. 10 in Eq. 11 and using the Green theorem:

$$\int_V \nabla\phi \cdot \nabla\phi d\mathbf{r} = \int_\Sigma \phi\nabla\phi \cdot d\boldsymbol{\sigma} - \int_V \phi\nabla^2\phi d\mathbf{r} \tag{13}$$

where the surface integral is zero, we obtain the following Hamiltonian:

$$H = -\frac{1}{8\pi}\int \left[\underbrace{-\phi\nabla^2\phi}_{\mathbf{E}^2_{\text{long}}} \underbrace{-\frac{\dot{\mathbf{A}}\cdot\dot{\mathbf{A}}}{c^2} - (\nabla\times\mathbf{A})\cdot(\nabla\times\mathbf{A})}_{\mathbf{E}^2_{\text{trans.}}+\mathbf{B}^2} \right] d\mathbf{r}$$
$$+\int \Psi_e^\dagger[c\alpha_i(-i\hbar\nabla_i - \frac{e}{c}A_i - \frac{e}{c}A_i^{\text{ext}}) + \beta m_e c^2)]\Psi_e d\mathbf{r} + \int \phi\rho d\mathbf{r} + \int \phi_{ext}\rho d\mathbf{r} \tag{14}$$

1.2.1 Relativistic Pauli-Fierz Hamiltonian in the length gauge

When investigating molecular systems, it is usually more convenient to apply a unitary transformation to the field modes, allowing for a direct coupling between the field operators and the molecular dipole. This transformation is known as the length-gauge transformation:

$$U = \exp\left(\frac{ie}{\hbar c}\mathbf{A}_{\text{dm}}(\vec{0})\cdot\mathbf{R}\right) \tag{15}$$

where $\mathbf{R} = \int d\mathbf{r}\Psi^\dagger\mathbf{r}\Psi$. Hamiltonian 26 in the main text can be transformed in the length-gauge form by application of Eq. 15:

$$H^l_{RPF} = U^\dagger H_{QED} U \tag{16}$$

followed by a rotation of the photonic coordinates associated with the mode $\mathbf{k}_{\text{dm}}$: $\tilde{U} = \exp\left(-i\frac{\pi}{2}\sum_{\vec{\epsilon}} a'^\dagger_{\text{dm},\vec{\epsilon}} a'_{\text{dm},\vec{\epsilon}}\right)$ which ensures the photonic part to be real. Transformation in Eq.16 induces a cancellation of the $\boldsymbol{\alpha}\cdot\mathbf{A}_{\text{dm}}$ term due to the change in the momentum. The light-matter coupling is now related to the molecular dipole.

1.3 Length gauge transformation

In the article, we presented the results of the application of the length gauge transformation. In this appendix, we provide a detailed derivation of such results. Since most of the terms of Hamiltonian 24 in the main text commute with the U operator, only two terms get modified,

the one involving the momentum p and the one involving the photon number operator $a_\tau^\dagger a_\tau$. Using the special case of the Becker-Campbell-Hausdorff formula:

$$\begin{aligned}&\int d\mathbf{r}\Psi^\dagger c\alpha^i p_i\Psi\\ &\longrightarrow \int d\mathbf{r}\Psi^\dagger c\alpha^i p_i\Psi - \left[\frac{ie}{\hbar c}\mathbf{A}_{\rm dm}(\vec{0})\cdot\mathbf{R}, \int d\mathbf{r}\Psi^\dagger c\alpha^i p_i\Psi\right].\end{aligned} \tag{17}$$

Let us consider the commutator only:

$$\frac{ie}{\hbar c}A_{j,\rm dm}\left[\int d^3r'\Psi^\dagger(\vec{r}')r^j\Psi(\vec{r}'), \int d\mathbf{r}\Psi^\dagger(\vec{r})c\alpha^i p_i\Psi(\vec{r})\right] \tag{18}$$

$$= \frac{ie}{\hbar c}A_j\int d^3r'\Psi^\dagger(\vec{r}')\Psi(\vec{r}')\underbrace{\left[r^j, p_i\right]}_{=i\hbar\delta_i^j}\int d\mathbf{r}\Psi^\dagger(\vec{r})c\alpha^i\Psi(\vec{r}) \tag{19}$$

$$= -eA_{j,\rm dm}\underbrace{\int d^3r'\Psi^\dagger(\vec{r}')\Psi(\vec{r}')}_{1}\int d\mathbf{r}\Psi^\dagger(\vec{r})\alpha^j\Psi(\vec{r}) \tag{20}$$

$$= -\int d\mathbf{r}\Psi^\dagger(\vec{r})eA_{j,\rm dm}\alpha^j\Psi(\vec{r}) \tag{21}$$

and therefore

$$\int d\mathbf{r}\Psi^\dagger c\alpha^i p_i\Psi \longrightarrow \int d\mathbf{r}\Psi^\dagger c\alpha^i\left(p_i + \frac{e}{c}A_{i,\rm dm}(\vec{0})\right)\Psi. \tag{22}$$

It is important to emphasize that the dipole approximation strongly simplifies the expression of the terms involving the momentum. The other noticeable fact is that the terms involving $\alpha^i A_i(\vec{0})$ cancels out (the translated momentum bring out a $+e\mathbf{A}_{\rm dm}$ term canceling out with the unmodified $-e\mathbf{A}_{\rm dm}$ term).

Let us now look at the modification of the photon number operator. The Becker-Campbell-Hausdorff formula implies that:

$$a'^\dagger_{{\rm dm},\vec{\epsilon}}a'_{{\rm dm},\vec{\epsilon}} \longrightarrow a'^\dagger_{{\rm dm},\vec{\epsilon}}a'_{{\rm dm},\vec{\epsilon}} - \left[\frac{ie}{\hbar c}\mathbf{A}_{\rm dm}(\vec{0})\cdot\mathbf{R}, a'^\dagger_{{\rm dm},\vec{\epsilon}}a'_{{\rm dm},\vec{\epsilon}}\right] + \ldots \tag{23}$$

The first commutator gives:

$$-\frac{ie}{\hbar c}\frac{C\mathbf{R}\cdot\vec{\epsilon}}{\sqrt{\omega_{\rm dm}}}\left[a'_{{\rm dm},\vec{\epsilon}}-a'^{\dagger}_{{\rm dm},\vec{\epsilon}}\right] \tag{24}$$

where $C=\sqrt{\frac{2\pi\hbar}{V}}$. The second commutator gives:

$$+\left(\frac{Ce\mathbf{R}\cdot\vec{\epsilon}}{\hbar c}\right)^2\frac{1}{\omega_{\rm dm}}. \tag{25}$$

Therefore, the Hamiltonian then reads:

$$\begin{aligned}
&\mathrm{H}^l_{RPF}= \\
&\int d\mathbf{r}\Psi^\dagger\{c\alpha^i\left(p_i-\frac{e}{c}A_{{\rm om},i}(\mathbf{r})-\frac{e}{c}A_{{\rm ext},i}(\mathbf{r})\right)+\beta m_e c^2\}\Psi \\
&+\frac{1}{2}\int d\mathbf{r}d\mathbf{r'}\Psi^\dagger(\mathbf{r})\Psi(\mathbf{r})\frac{1}{|\mathbf{r}-\mathbf{r'}|}\Psi^\dagger(\mathbf{r'})\Psi(\mathbf{r'}) \\
&+\sum_{\vec{\epsilon}}\hbar\omega_{\rm dm}\{a'^{\dagger}_{{\rm dm},\vec{\epsilon}}a'_{{\rm dm},\vec{\epsilon}}-\frac{ie}{\hbar c}\frac{C\mathbf{R}\cdot\vec{\epsilon}}{\sqrt{\omega_{\rm dm}}}\left[a'_{{\rm dm},\vec{\epsilon}}-a'^{\dagger}_{{\rm dm},\vec{\epsilon}}\right] \\
&+\left(\frac{Ce\mathbf{R}\cdot\vec{\epsilon}}{\hbar c}\right)^2\frac{1}{\omega_{\rm dm}}+\frac{1}{2}\} \\
&+\sum_{\tau}\hbar\omega_\tau\left(a'^{\dagger}_\tau a'_\tau+\frac{1}{2}\right).
\end{aligned} \tag{26}$$

The final Hamiltonian in length gauge appears as:

$$\begin{aligned}
\mathrm{H}^l_{RPF}=&\int d\mathbf{r}\Psi^\dagger h\Psi \\
&+\frac{1}{2}\int d\mathbf{r}d\mathbf{r'}\Psi^\dagger(\mathbf{r})\Psi(\mathbf{r})\frac{1}{|\mathbf{r}-\mathbf{r'}|}\Psi^\dagger(\mathbf{r'})\Psi(\mathbf{r'}) \\
&+\sum_{\vec{\epsilon}}\hbar\omega_{\rm dm}\left[a^{\dagger}_{{\rm dm},\vec{\epsilon}}a_{{\rm dm},\vec{\epsilon}}-\frac{e}{\hbar c}\frac{C\mathbf{R}\cdot\epsilon_\tau}{\sqrt{\omega_{\rm dm}}}(a_{{\rm dm},\vec{\epsilon}}+a^{\dagger}_{{\rm dm},\vec{\epsilon}})\right. \\
&\left.+\left(\frac{Ce\mathbf{R}\cdot\epsilon_\tau}{\hbar c}\right)^2\frac{1}{\omega_{\rm dm}}+\frac{1}{2}\right]
\end{aligned} \tag{27}$$

where h is :

$$h=c\alpha^i\left(p_i-\frac{e}{c}A_{{\rm om},i}(\mathbf{r})-\frac{e}{c}A_{{\rm ext},i}\right)+\phi_{\rm ext}+\beta m_e c^2 \tag{28}$$

where $C = \sqrt{\frac{2\pi\hbar}{V}}$. Here, $a_{\mathrm{dm},\vec{\epsilon}}$, $a^{\dagger}_{\mathrm{dm},\vec{\epsilon}}$ have been relabeled to emphasize that, due to the length gauge transformation, the latter are modified. In fact, $\langle a^{\dagger}_{\mathrm{dm},\vec{\epsilon}} a_{\mathrm{dm},\vec{\epsilon}}\rangle$ does not coincide with the number of photons related to the mode $\mathbf{k}_{\mathrm{dm}}$ anymore.[7] This Hamiltonian is the analogous of the standard Pauli-Fierz Hamiltonian usually applied in non-relativistic polaritonic chemistry,[8–10] where molecular orbitals are replaced by molecular spinors. Equation 27 has an apparent origin dependence, coming from the presence of the dipole operator $\mathbf{R}$. This problem can be solved by the coherent state transformation $U_c = \Pi_{\vec{\epsilon}} \exp\left(z_{\vec{\epsilon}} a^{\dagger}_{\mathrm{dm},\vec{\epsilon}} - z^{*}_{\vec{\epsilon}} a_{\mathrm{dm},\vec{\epsilon}}\right)$ with $z_{\vec{\epsilon}} = \frac{e\sqrt{2\pi}}{c\sqrt{\hbar\omega_{\mathrm{dm}} V}} \langle \mathbf{R} \cdot \vec{\epsilon}\rangle$.[10] Applying this transformation to the Relativistic-Pauli-Fiertz (RPF) Hamiltonian, we obtain:

$$
\begin{aligned}
\mathrm{H}^{l}_{RPF} = & \int d\mathbf{r} \Psi^{\dagger} h \Psi \\
& + \frac{1}{2} \int d\mathbf{r} d\mathbf{r}' \Psi^{\dagger}(\mathbf{r}) \Psi(\mathbf{r}) \frac{1}{|\mathbf{r} - \mathbf{r}'|} \Psi^{\dagger}(\mathbf{r}') \Psi(\mathbf{r}') \\
& + \sum_{\vec{\epsilon}} \hbar\omega_{\mathrm{dm}} \left(a^{\dagger}_{\vec{\epsilon}} a_{\vec{\epsilon}} + \frac{1}{2} \right) + \left(\frac{e}{c} \sqrt{\frac{2\pi}{V}} \vec{\epsilon} \cdot [\mathbf{R} - \langle \mathbf{R} \rangle] \right)^{2} \\
& - \sqrt{\hbar\omega_{\mathrm{dm}}} \left(\frac{e}{c} \sqrt{\frac{2\pi}{V}} \vec{\epsilon} \cdot [\mathbf{R} - \langle \mathbf{R} \rangle] \right) \left(a_{\vec{\epsilon}} + a^{\dagger}_{\vec{\epsilon}} \right),
\end{aligned}
\tag{29}
$$

2 Excited states properties of CuH and AgH

In Fig.SI 1, we report the dispersion of the CuH excitation energies with respect to the cavity frequency. Also in this case, despite significantly smaller than the one observed for AuH in Fig. 6 in the main text, we detect an observable Rabi-splitting. This is expected since the Cu atom is much lighter than gold and relativistic effects, including the spin-orbit coupling, are significantly smaller. In this case, the energy difference between the $\Omega = 0$ and $\Omega = 1$ state is 20 times smaller than for AuH (~ 0.002 eV).

Similar results can be observed also in Fig.SI 2 for AgH. In this case, the observed behavior is somehow intermediate between the one of CuH and the one of AuH, consistently with the fact that Ag is heavier than Cu but lighter than Au. For this system the degeneracy between

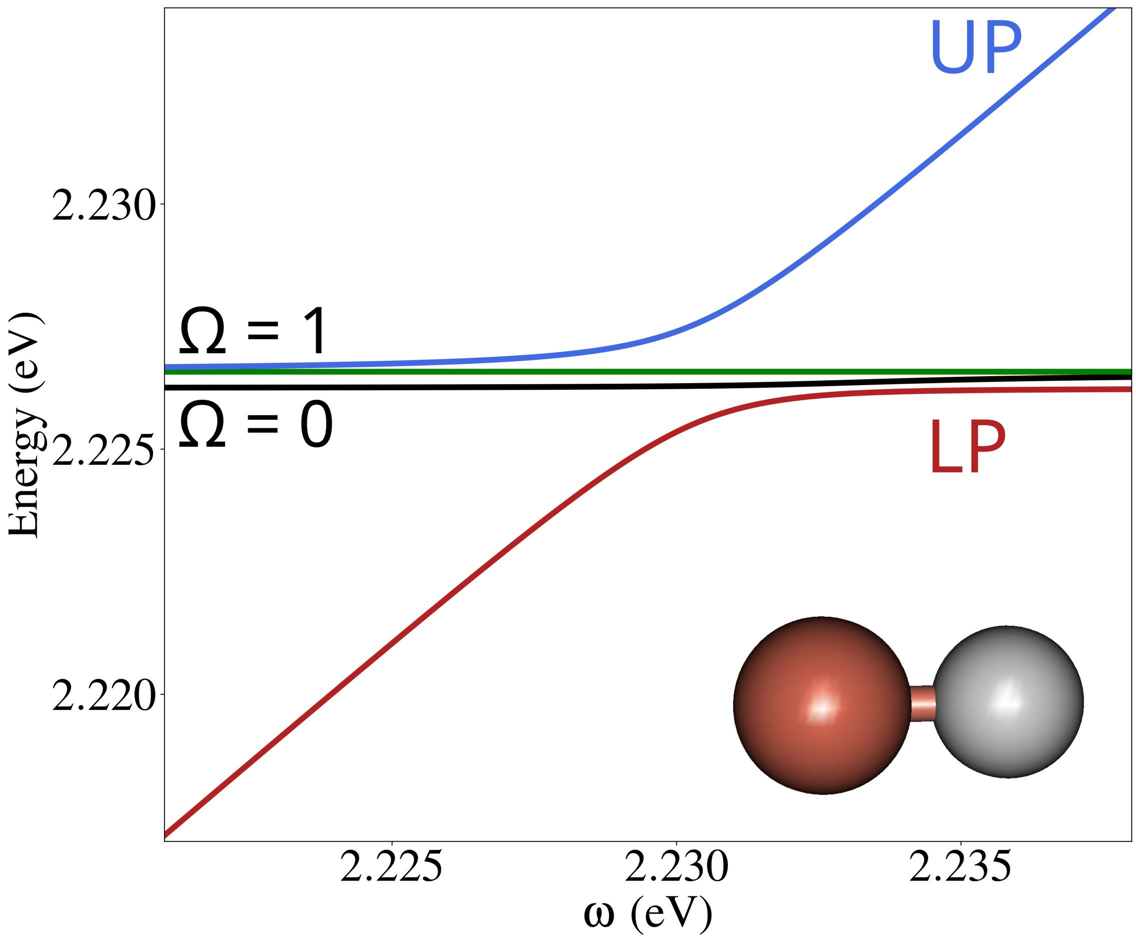

Figure 1: Excitation energies evaluated with linear response Pol-DHF as a function of the cavity frequency for CuH.

the three states is slightly lifted due to the 4-component treatment. However, contrary to AuH the energy difference between the $\Omega = 0$ and $\Omega = 1$ state is significantly smaller ($\sim$ 0.009 eV). Notice that for both CuH and AgH, the Rabi-splitting is larger than such energy difference.

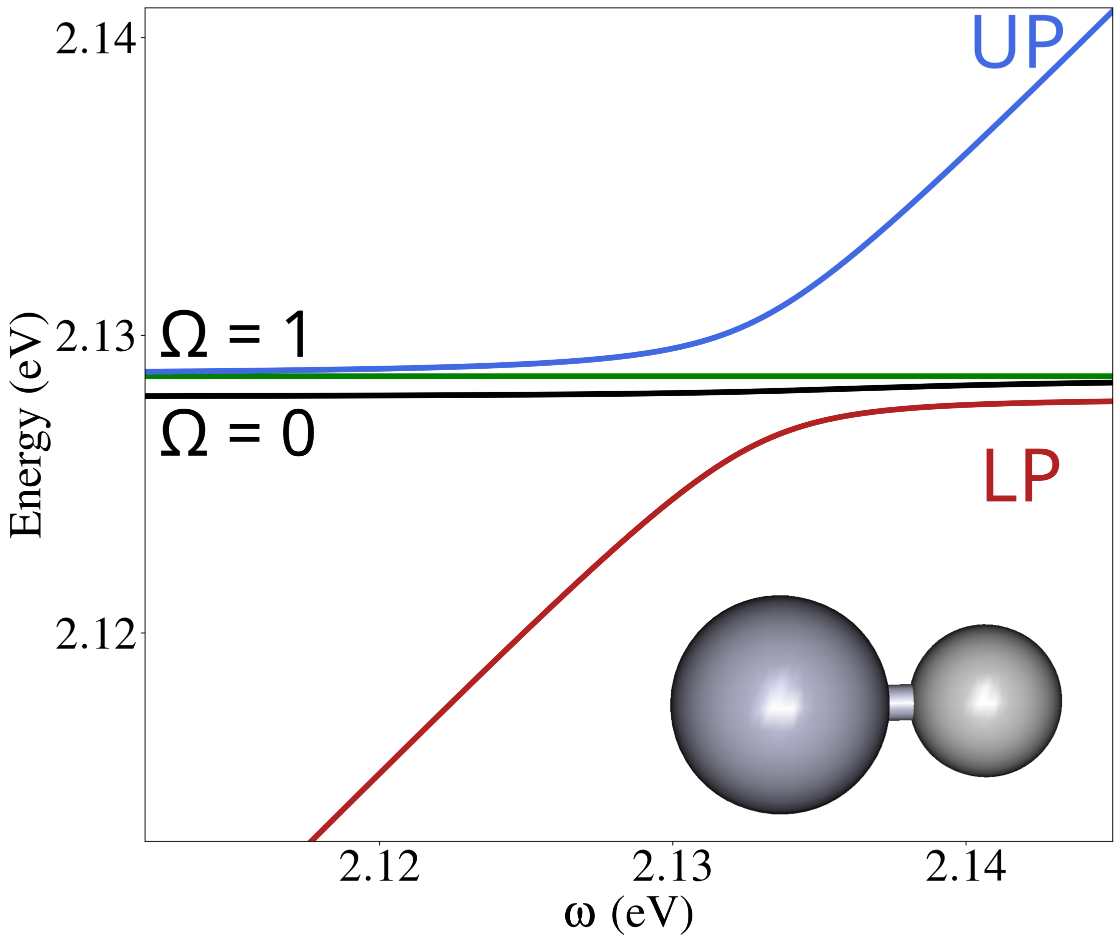

Figure 2: Excitation energies evaluated with linear response Pol-DHF as a function of the cavity frequency for AgH.

3 Effect of using an uncontracted basis set

As mentioned in the manuscript, in this work, for computational reasons we have used a contracted basis set, though it is not recommended when using a 4-component method. We have performed calculations with an uncontracted basis set on the smallest hydride CuH in order to assess the impact on our results. While the ground state properties have not been significantly affected by this change, the excited states properties were slightly more affected. Indeed, using an uncontracted basis set tends to amplify the effect of the spin-orbit

coupling. Therefore, for a system with a low spin-orbit coupling, the Rabi-Splitting while be smaller since it depends on the spin-orbit coupling. However, it is important to stress that the qualitative behavior remains the same and the effect of the spin-orbit coupling and the Rabi-Splitting is comparable for systems like CuH where relativistic effects are expected to be less important.

Table 1: Ground state total energies calculated at the DHF and Pol-DHF level for CuH with an uncontracted basis set. $\Delta E_{Pol} = E_{Pol\text{-}DHF} - E_{DHF}$.

molecule	DHF (Hartree)	Pol-DHF (Hartree)	ΔE_{Pol} (eV)
CuH	-1653.7276833807055	-1653.7333641604048	-0.1546

Table 2: Energy differences between DHF and DHF-Gaunt(-Breit) level for CuH with an uncontracted basis set.

molecule	No Pol		Pol	
	Gaunt (eV)	Breit (eV)	Gaunt (eV)	Breit (eV)
CuH	20.2548	-1.83556	20.2547	-1.83555

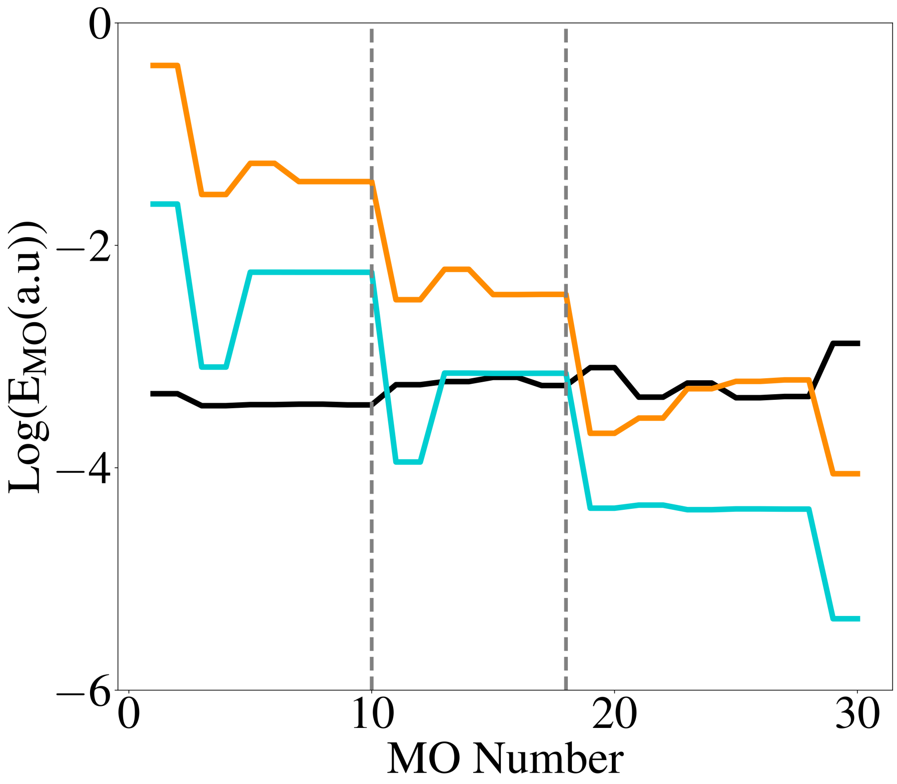

Figure 3: Absolute contribution of Polaritonic (Black), Gaunt (Orange) and Breit (Cyan) terms on the various molecular orbitals of CuH with and uncontracted basis set. Three zones have been represented on the graph, the one on the left corresponds to the core region, the middle one is an area where Gaunt, Breit and Polaritonic contributions are comparable, and the area on the right to the valence region.

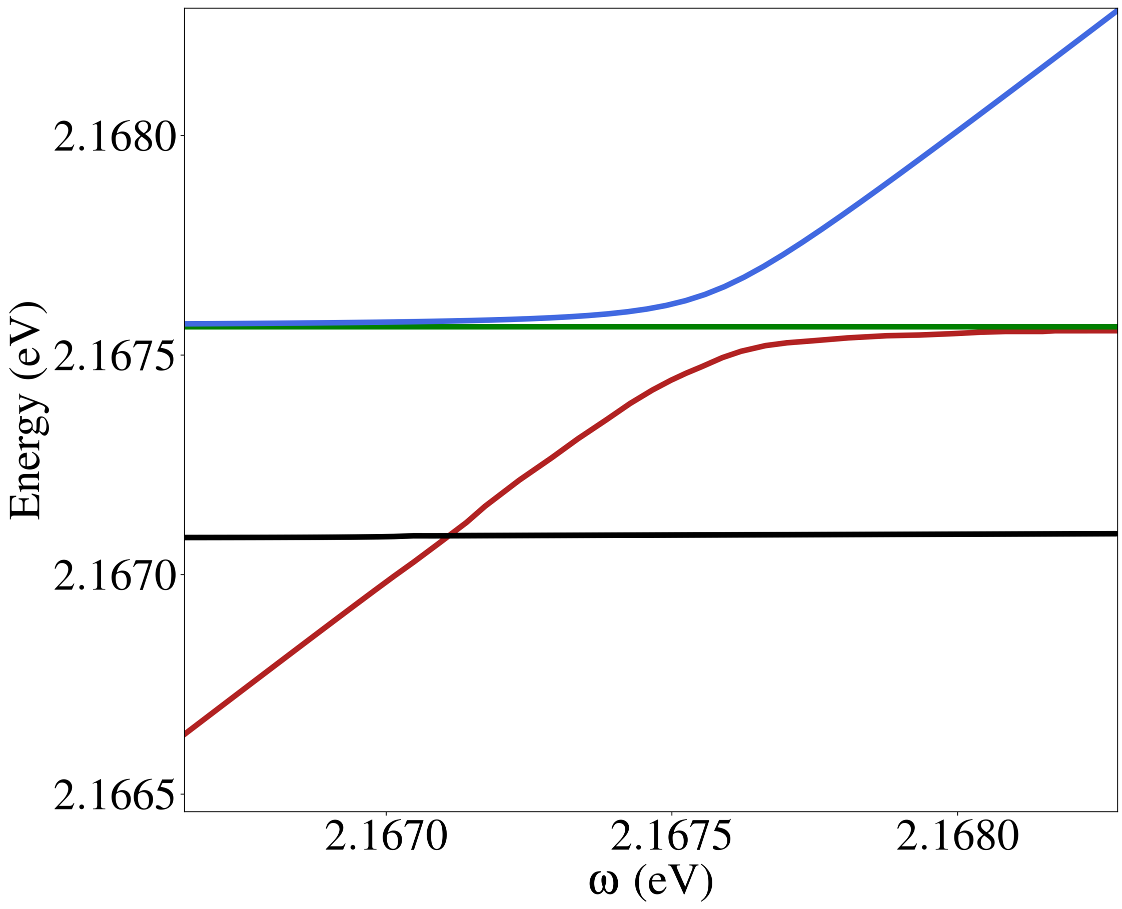

Figure 4: Excitation energies evaluated with linear response Pol-DHF as a function of the cavity frequency for CuH with an uncontracted basis set.

References

(1) Cohen-Tannoudji, C.; Dupont-Roc, J.; Gryndberg, G. *Photons and Atoms : Introduction to quantum electrodynamics*; John Wiley & Sons, Ltd, 1997.

(2) Reiher, M.; Wolf, A. *Relativistic Quantum Chemistry*; John Wiley & Sons, Ltd, 2014.

(3) Jackson, J. D. From Lorenz to Coulomb and other explicit gauge transformations. *American Journal of Physics* **2002**, *70*, 917–928.

(4) Stewart, A. M. Vector potential of the Coulomb gauge. *European Journal of Physics* **2003**, *24*, 519.

(5) Peskin, M.; Schroeder, D. *An Introduction To Quantum Field Theory*; Frontiers in Physics; Avalon Publishing, 1995.

(6) Tong, D. Lectures on Quantum Field Theory. 2006; `https://www.damtp.cam.ac.uk/user/tong/qft.html`.

(7) Flick, J.; Schäfer, C.; Ruggenthaler, M.; Appel, H.; Rubio, A. Ab Initio Optimized Effective Potentials for Real Molecules in Optical Cavities: Photon Contributions to the Molecular Ground State. *ACS Photonics* **2018**, *5*, 992–1005.

(8) Ruggenthaler, M.; Flick, J.; Pellegrini, C.; Appel, H.; Tokatly, I. V.; Rubio, A. Quantum-electrodynamical density-functional theory: Bridging quantum optics and electronic-structure theory. *Phys. Rev. A* **2014**, *90*, 012508.

(9) Wang, X.; Ronca, E.; Sentef, M. A. Cavity quantum electrodynamical Chern insulator: Towards light-induced quantized anomalous Hall effect in graphene. *Phys. Rev. B* **2019**, *99*, 235156.

(10) Haugland, T. S.; Ronca, E.; Kjønstad, E. F.; Rubio, A.; Koch, H. Coupled Cluster Theory for Molecular Polaritons: Changing Ground and Excited States. *Phys. Rev. X* **2020**, *10*, 041043.
